# Molecular Advances to Combat Different Biotic and Abiotic Stresses in Linseed (*Linum usitatissimum* L.): A Comprehensive Review

**DOI:** 10.3390/genes14071461

**Published:** 2023-07-17

**Authors:** Shruti Paliwal, Manoj Kumar Tripathi, Sushma Tiwari, Niraj Tripathi, Devendra K. Payasi, Prakash N. Tiwari, Kirti Singh, Rakesh Kumar Yadav, Ruchi Asati, Shailja Chauhan

**Affiliations:** 1 Department of Genetics and Plant Breeding, College of Agriculture, Rajmata Vijayaraje Scindia Krishi Vishwa Vidyalaya, Gwalior 474002, India; shruti1paliwal@gmail.com (S.P.); sushma2540@gmail.com (S.T.); kirti1751996@gmail.com (K.S.); rakeshyadav07081996@gmail.com (R.K.Y.); ruchiasati.95@gmail.com (R.A.); chauhanshailu24@gmail.com (S.C.); 2 Department of Plant Molecular Biology and Biotechnology, College of Agriculture, Rajmata Vijayaraje Scindia Krishi Vishwa Vidyalaya, Gwalior 474002, India; tiwarisprakashn051194@gmail.com; 3 Directorate of Research Services, Jawaharlal Nehru Krishi Vishwa Vidyalaya, Jabalpur 482004, India; 4 All India Coordinated Research Project on Linseed, Jawaharlal Nehru Krishi Vishwa Vidyalaya, Regional Agricultural Research Station, Sagar 470001, India; dpayasi@gmail.com

**Keywords:** *Linum usitatissimum* L., biotic and abiotic stresses, integrated breeding, omics, transgenic

## Abstract

Flax, or linseed, is considered a “superfood”, which means that it is a food with diverse health benefits and potentially useful bioactive ingredients. It is a multi-purpose crop that is prized for its seed oil, fibre, nutraceutical, and probiotic qualities. It is suited to various habitats and agro-ecological conditions. Numerous abiotic and biotic stressors that can either have a direct or indirect impact on plant health are experienced by flax plants as a result of changing environmental circumstances. Research on the impact of various stresses and their possible ameliorators is prompted by such expectations. By inducing the loss of specific alleles and using a limited number of selected varieties, modern breeding techniques have decreased the overall genetic variability required for climate-smart agriculture. However, gene banks have well-managed collectionns of landraces, wild linseed accessions, and auxiliary Linum species that serve as an important source of novel alleles. In the past, flax-breeding techniques were prioritised, preserving high yield with other essential traits. Applications of molecular markers in modern breeding have made it easy to identify quantitative trait loci (QTLs) for various agronomic characteristics. The genetic diversity of linseed species and the evaluation of their tolerance to abiotic stresses, including drought, salinity, heavy metal tolerance, and temperature, as well as resistance to biotic stress factors, viz., rust, wilt, powdery mildew, and alternaria blight, despite addressing various morphotypes and the value of linseed as a supplement, are the primary topics of this review.

## 1. Introduction

Flaxseed (*Linum usitatissimum* L.) is one of the oldest cropsand has been farmed for a long time. In Latin, its name translates to “extremely beneficial” crop, presumably because it is a source of three main bioactive components, i.e., α-linolenic acid, lignans, and dietary fibre [1]. This important crop is an annual herb that produces golden-yellow- to reddish-brown-coloured small, flat seeds. In the beginning, the crop was introduced to the US, predominantly to create textile fibres [2]. The stem creates robust, long-lasting, high-quality fibres, which is perhaps one of the reasons that it was named flaxseed. Flax produced from this crop was primarily utilised in the production of textiles (linen) and paper industries. The name linseed is generally used in terms of oil production and other byproducts, like animal feed [3,4].

*L. usitatissimum* is the only cultivated species among the roughly 200 species of the genus *Linum*, and it is farmed for the essential oils in its seeds and for the fibre in the stem that it produces [5,6]. Linseed domestication started with the selection of traits and more effective self-fertilisation, while Mansby et al. [7] claimed that out-crossing was of greater value. Most linseed variants are created by crossing within the gene pool of *L. usitatissimum* [8,9].

The importance of *L. usitatissimum* as a multifunctional crop is demonstrated by the fact that various plant parts have various economic uses. Flax fibres, which have two to three times the strength in comparison to cotton fibres, and both fibres can be combined to make linen [10]. The two most popular varieties of flax are brown and golden (or yellow). Both contain equivalent amounts of short-chain fatty acids withhigh nutritional profiles. Whole flaxseed meal, powdered flaxseed, flaxseed oil extracted from the seeds, and partially defatted flaxseed meal are the four most frequent ways to consume flaxseed [11]. Flax “milk” is a new product in the market and establishing itself because it is free from lactose, along with cholesterol. It is a big source of omega fatty acids, fibre, and protein. Flax milk may be a fantastic substitute for dairy milk. It is great for individuals who are intolerant to gluten, nuts, or soy, and it is healthier than almond milk [12,13,14,15,16]. Because of the possible health advantages, particularly those linked to its biochemically active elements, flaxseed has gained significant interest in the area of dietary and health research over the last 20 years [17]. Flaxseeds have been used traditionally in many Ayurvedic preparations since ancient times [17]. In Ayurveda, they are used for “vata”- and “pitta”-related disorders [12]. It is also essential in processes such as virechana (pharmacologic laxative therapy). Hippocrates used flax to treat abdominal pain [18]. Flaxseed is an excellent dietary supplement that deserves more research [13]. On average, flaxseed contains about 30–40% oil, is high in protein at 20–25%, and contains 20–28% total fibre and minerals [1]. Seed oil is nutritionally superior because of its high amounts of α-linolenic acid, omega-3 fatty acid, omega-6 fatty acids, and vitamins A, B, D, and E [15]. These small seeds are believed to aid digestion and lower the risk of cardiovascular disease and especially type-2 diabetes [16]. However, some people reject flaxseed as a dietary supplement because of concerns about anti-nutritional factors [10,19].

For a breeding programme to be successful, diversity is a key component [20,21,22]. Initially, genetic variation evaluations relied on morphological and biochemical indicators, such as isozymes [23,24]. Nevertheless, phenotypic characteristics are labour- and time-intensive, as well as sensitive to environmental factors. Biochemical markers may also not be useful for variation analysis among crop genotypes due to their alteration with environmental conditions [25]. DNA-based markers are stretches of nucleotides and are not influenced by environmental conditions [26]. Random amplified polymorphic DNA (RAPD), inter simple sequence repeat (ISSR), simple sequence repeat (SSR), amplified fragment length polymorphism (AFLP), sequence-tagged site (STS), and single-nucleotide polymorphism (SNP) are among the various molecular markers being used for agricultural plant genetic variation analysis [27]. Except for SNPs, other markers do not require in-depth genomic information and are simpler, less costly, and less labour-intensive than other DNA marker approaches [10]. Some studies are available on applications of markers for diversity analysis in linseed. Such markers include isozymes [28], RAPD [29,30,31,32,33], AFLP [34,35,36,37], ISSR [38,39,40], and SSR [41,42]. 

The basis of linseed genetic improvement and cultivar development is the conventional breeding technique, which has resulted in the release of new cultivars with long-lasting disease resistance, improved yield stability, and better agronomic performance [43]. However, molecular breeding [44,45] has also been applied for this purpose. A restricted genetic base was employed to develop a Canadian linseed variety [46,47,48]. The absence of related species for incorporating novel genes has hampered yield and quality innovations, hindering linseed competitiveness [11].

In the years to come, climate change may put enormous pressure on the breeding society to breed varieties [49] having adjustableproperties underadverse climatic conditions. For any crop to be improved or adjusted to changing environmental conditions or market demands, access to genetic variability is a must, and the availability of diverse germplasm lines of the targeted crop plays a significant role in it [10,50,51,52]. A variety of germplasms, including wild relatives, landraces, commercial, extinct, and elite varieties, pure and breeding lines, mutants, polyploids, and hybrids, are found in gene bank collections [11] and can be exploited for the improvement of the crop. There are 46,513 linseed/flax accessions documented in global germplasm collections, of which *L. bienne* (the wild ancestor of farmed flax) is only intermittently represented in gene banks [53]. Breeders can use these plant genetic resources of linseed for the improvement of linseed crops with potential climate adaptations [54,55]. Following the preliminary assessments of flax germplasm collections, subsequent evaluations for different characteristics associated with tolerance to different biotic and abiotic stress factors were carried out [56,57], alongside the present concentration on the germplasm screening of single-gene attributes, such as disease resistance [58]. As a result, the extensive diversity found in linseed germplasm collections, along with data on characterisation and assessment, is a potential source that is worth using in breeding for abiotic and biotic stresses. The current review focuses on all the advancements made in the flax research programme to overcome these stresses.

## 2. Abiotic Stress 

To achieve agricultural sustainability, it is critical to develop and nurture products that are tolerant to rising abiotic pressures caused by climate variability [49]. Numerous abiotic and biotic stressors that constantly threaten plants have an impact on their outputs. Through intricate endogenous signalling networks and numerous modifications, the plant reacts to these stressors. Under these environmental circumstances, the plant’s output and reproductive success are determined by the interactions between these networks. Linseed, like other crops, if subjected to a variety of abiotic stresses, might have reduce yields [59]. The different abiotic stresses influencing linseed are summarised in the following subsections.

### 2.1. Drought Tolerance

Drought is considered one ofthe most pervasive and harmful abiotic factors that impact agricultural output. Drought, on average, affects crop growth, production, and quality by more than 50% [60,61,62,63]. By 2050, nearly fifty percent of land that is usable for agriculture is anticipated to have disastrous effects on plant development [64]. Soil moisture deficiency can have a substantial impact on the flax output capacity, oil quantity and fatty acid arrangement, and fibre quality indicators [65,66,67]. 

According to Hu and Xiong [68], drought causes normal metabolism to be disrupted by decreased leaf growth, oxidative damage, increased membrane lipid peroxidation, leaf senescence, and abscission. Flax can tolerate drought conditions more effectively than numerous other oil and food crops due to its hardiness [69]; however, flax plants lose a lot of water due to their high transpiration coefficients, which range from 787 to 1093 [70]. To generate optimal yields, fibre flax requires at least 600–650 mm of yearly precipitation, with at least 110–150 mm falling during the vegetative season [71,72]. Since drought is an unpredictable and irregular environmental event, genotypes with high yield potential and drought tolerance may be selected [10].

To overcome these limitations and enhance flax yields, research activities based on conventional breeding and later transgenic approaches were initiated [73]. Understanding the adaptation processes and identifying the underlying genes, markers, and QTLs might help to improve the genetics and production of linseed in semi-arid and arid areas because drought tolerance is a complicated polygenic trait [49]. A limited number of studies have been published identifying the accessions of flax that are resistant to drought [74,75,76] and genome-wide analyses of drought-induced gene expression [72]. Linseed has a shallow root system as compared to other oilseed crops, such as safflower, rapeseed, and sunflower. Therefore, understanding the root system architecture is crucial for improving flax’s ability to absorb water. Many crops, including rice, wheat, and maize, have recently demonstrated the significance of specific root characteristics for efficient water and nutrient absorption under water-stress conditions [77,78,79,80], but knowledge is still somewhat limited in flax. Among different morpho-physiological characteristics, plant height, biomass, seed colour, lignin content, seed yield, leaf absolute water content, and leaf relative water content have been found to be associated with drought tolerance in linseed [81,82].

Plants have many adaptations for surviving in conditions of drought and/or water shortage [83,84]. It is crucial to comprehend these processes in order to create agricultural plants that are resistant to such circumstances [85]. Quéro et al. [86] tried to investigate the processes underlying b-aminobutyric acid (BABA)-induced drought adaptation in plants. BABA causes a reorganisation of the solute content in flax leaves that results in an increased accumulation of proline and nonstructural carbohydrates, as well as a drop in inorganic solutes, according to metabolomic and ionomic profiling of the leaves [59]. In one of the previous studies, it was discovered that BABA therapy caused alterations that made flax plants more resistant to drought stress [86]. Under various irrigation regimes, Ansari et al. [87] investigated the relationship between the crop and mycorrhizal fungi. In a recent study conducted by Liu et al. [11], it was discovered that, in contrast to various plant species, flax mycorrhizal fungus may promote development under both stress and non-stress conditions.

Shivaraj et al. [59] explained the effects of various amounts of titanium dioxide (TiO_2_) at the nanoscale (10–25 nm) on flax development under stress and non-stress circumstances. It was discovered that TiO_2_ at modest concentrations facilitated the development of flax in water-scarce environments. Additionally, flax plants treated with nano-TiO_2_ at a concentration of 100 mg L^−1^ contained higher seed oil and protein contents. As a result, it has been concluded that the application of nano-TiO_2_ particles at modest concentrations can reduce the harm caused by drought stress to flax plants as well as increase drought tolerance. Crops that are resistant to duress are developed using both forward and backward genetic methods. In the same setting, the drought-responsive element binding protein 2A (*DREB2A*) gene was inserted into the plant genome to create a flax cv. Blanka cell line that is drought-tolerant [73].

### 2.2. Salinity Tolerance

Numerous variables, such as obscene irrigation, rock weathering, little precipitation, ion exchange, high surface evaporation, and poor cultivation practices, have contributed to the exponential increase in soil salinity in recent years [88,89,90]. According to estimates from Shrivastava and Kumar [91], greater than fifty percent of arable land will be salinised by around 2050. Currently, salty conditions impact around 33% of irrigated land and 20% of all cultivated land. According to Dubey et al. [88], soil salinity–alkalinity in flax causes delayed germination and emergence, reduced seedling survival, erratic crop development, and decreased yield. In a few investigations, flax germplasm was tested for resistance to salinity–alkalinity stressors, and salinity-tolerant lines were found based on biomass, germination, seedling traits, and the K^+^/Na^+^ ratio [92,93,94,95,96]. Wu et al. [97] discovered genes in flax that increase the root length, improve membrane damage, and alter the ion distribution to confer salt tolerance. Flax may be cultivated on agricultural terrain where other crops cannot since it can withstand pH levels up to 9.

All agricultural plants face genuine threats from rising soil salinity and alkalinity, among which alkaline-salt stress is more harmful than neutral-salt stress. Considering this, understanding the processes that control plant resistance to saline–alkaline stress has emerged as a hotly debated area of plant science. The impact of these circumstances on the sprouting of the flax plant was examined by Guo et al. [98]. In three experimental circumstances, they discovered that germination decreased with a rise in the ionic concentrations for each of the 10 common flax genotypes they examined. As anticipated, it was discovered that low-concentration treatments with neutral salt and alkaline salt had little impact on germination in all varieties [11].

All varieties of plants responded the most negatively to alkaline-salt stress, and higher concentrations of this treatment completely prevented germination. When five linseed genotypes were developed after being treated with neutral salt and alkaline salt, it was discovered that they had greater salt tolerance [98]. Digital gene expression in flax plants under alkaline-salt stress, neutral-salt stress, and alkaline stress was examined by Yu et al. [99]. In their experiment, it was discovered that under neutral-salt stress, carbohydrate metabolism was impacted, while under alkaline-salt stress, photosynthesis and thereaction to biotic stimuli were badly compromised. In some of the studies, the differential expression of important factors was implicated in responses to abiotic stressors, like WRKY, abscisic acid (ABA), ion channels, and mitogen-activated protein kinase (MAPKK) [10,59]. More of these differentially expressed genes were triggered by alkaline-salt stress than by alkaline stress or neutral-salt stress, indicating that a greater number of genes are involved in regulating the alkaline-salt stress pathway.

Salinity stress has a molecular foundation that is still poorly known. Therefore, Yu et al. [100] used deep sequencing to analyse small RNAs and the degradome in samples that had been exposed to three stress conditions in order to understand the genetic basis of salinity tolerance in flax. Small RNA target genes that control reactions to cues were discovered to be induced. Using transcriptome analysis and degradation genome sequencing, 29 showed opposite expression patterns. The development of climate-smart flax is progressingforall stressors, and 243 miRNA–target combinations have been identified. Two miRNAs, miR398a and miR530, have been reported to be linked to salt stress tolerance in flax. Furthermore, in a separate experiment, Wei et al. [101] discovered that flax seedlings of HIZ019, YOI254, and Tianxin3 had higher salt tolerance because of some important biochemical alterations in flax seedlings under salt stress.

### 2.3. Heavy Metal Stress Tolerance

Heavy metal buildup in vegetation is caused by heavy metal contamination of the earth. Heavy metals in the dietary chain are consequently biomagnified. One such heavy metal that can be detrimental to both plants and animals, even in minute quantities, is cadmium. Plants have evolved specialised regulatory mechanisms that allow them to bind metals to compounds like phytochelatins and metallothionein to create complexes (metallothionein group III). Numerous studies have demonstrated that linen clothing tolerates cadmium pollution well. Even though the toxic effect of cadmium restricts its use for both food and medicine, the use of metal-accumulating plants for the phytoremediation of contaminated soils opens a novel and hopeful path towards improving the resilience of their cultivars and variants to Cd stress [59]. Over the past ten years, extensive research has been conducted on the mechanisms that drivecadmium tolerance in plants. High Cd concentrations reduced root development and, to different degrees, increased the generation of membrane permeability, hydrogen peroxide (H_2_O_2_), protein oxidation, and lipid peroxides. Additionally, it was discovered that there had been a substantial change in the effectiveness of antioxidant and scavenging enzymes. 

The impact of salicylic acid on the antioxidant defence system in flax plants was examined by Belkadhi et al. [102]. Salicylic acid, on the other hand, was discovered to lessen the toxic effects of Cd on the lipid composition of membranes, the antioxidant system, and root development [11]. Belkadhi et al. [103] discovered that a preliminary treatment with salicylic acid preferentially safeguarded plastidial lipids due to greater amounts of polyunsaturated fatty acids in flax. These findings imply that under Cd stress, salicylic acid-pretreated flax plantlets had increased membrane integrity. Salicylic acid boosts root antioxidant mechanisms in flax. Kaplan et al. [104] investigated the effects of cadmium stress on the fatty acid composition of flax in the presence of mycorrhizal fungi. They discovered that seeds from plants cultivated in mycorrhizal fungi contained more unsaturated (18:1, 18:2, and 18:3) fatty acids overall. At 15 ppm of Cd, these impacts became more obvious (the amounts of 18:1, 18:2, and 18:3 were increased by 169, 370, and 150%, respectively). These findings indicate that once the level of Cd in seeds hits a certain level, this heavy metal increases the effectiveness of the enzymes that control the transformation of saturated fatty acids into unsaturated fatty acids.

Although zinc (Zn) is a crucial ingredient needed for the healthy growth and development of plants, too much of it can also be harmful. Significant attempts have been made to comprehend the tolerance of plants to zinc in flax. Grant et al. [105] discovered that flax cultivars are better at transferring Zn through shoots to seeds than Cd. The use of plant species for phytoremediation is determined by the transfer of metal ions to seeds. Plants are also using a variety of biotechnological techniques to limit the absorption or transfer of the metal ions in plant cells. Smykalova et al. [106] investigated how flax varieties could collect and move Ca and Zn in an in vitro culture made from hypocotyl tissues. The flax varieties discovered by Smykalova et al. [106] are those that are tolerant to zinc, and increasing the amount of zinc and decreasing the amount of cadmium in the kernel will be crucial for increasing the nutritional value of linseed.

The toxic effects of heavy metals like copper (Cu), zinc (Zn), cadmium (cd), nickel (Ni), lead (Pb), chromium (Cr), arsenic (As), and cobalt (Co) on seed germination in various varieties of flax have been assessed in another important research conducted by Soudek et al. [107]. Various heavy metals manifested their harmful effects in the following order: As > Cu > Cd > Co > Cr > Ni > Pb > Zn. In fact, a lot of these studies point to the effective use of flax’s potential for phytoremediation, especially when cultivated for fibre production. The growing of flax on heavy-metal-contaminated soils may be made easier with a thorough grasp of the mechanisms underlying heavy metal tolerance [59].

### 2.4. Cold Stress Tolerance

Many cool-season perennial crops’ outputs are influenced by two significant physiological processes: vernalisation and photoperiodism. Darapuneni et al. [108] examined the flowering response of various flax genotypes under two photoperiod and vernalisation conditions in growth chamber research. The findings imply that the photoperiod, vernalisation, and genetics significantly influence early blooming in flax. For frigid climate regions like the Upper Midwest of the US and Canada, the early-blooming trait is more important. However, owing to the hot spring and summer temperatures in some places, such as Texas, flax is produced in the autumn. Flax varieties demonstrated genotypic interactions with the photoperiod and vernalisation.

In particular, Texas flax genotypes of the winter variety were susceptible to both vernalisation and blooming photoperiods. The flowering times of most other spring-grown flax genotypes were unchanged by the vernalisation treatments, whereas Texas genotypes postponed anthesis for 7 days or longer in unvernalised seedlings [10]. The majority of other genotypes were unaffected by circadian rhythms (photoperiodism)in vernalised seedlings, while Texas cultivars delayed anthesis for a period of twelve days or longer under vernalised and short-day conditions. Screening for vernalisation and photoperiodic sensitivity in Texas genotypes, as well as the introgression of these characteristics into newly adopted spring-grown genotypes, is required for the development of high-yielding flax genotypes for production sites in the southern Great Plains [59].

### 2.5. Heat Tolerance

**Figure 1:**
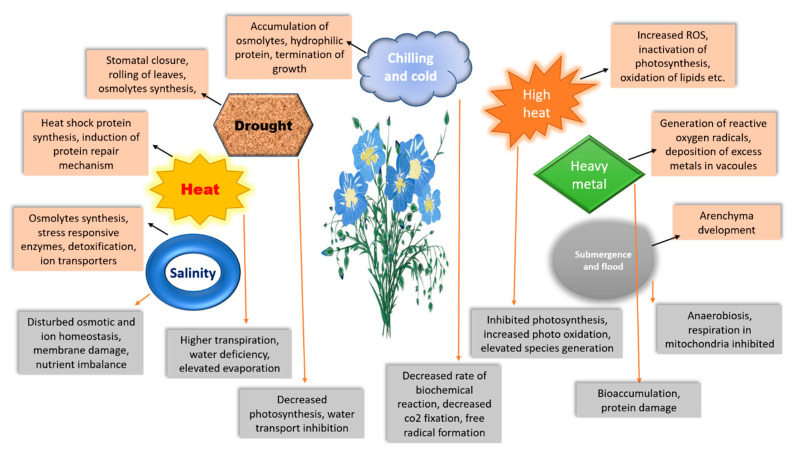
Schematic representation of abiotic stress regulation mechanisms by plants. The orange boxes represent coping mechanisms, while grey boxes represent disturbances in plant physiological response.

Particularly in tropical and subtropical climates, heat stress negatively impacts physiological processes, development (Figure 1), growth, and yield [109]. According to several studies [110,111,112,113], a prolonged duration of heat stress (40 °C for 5–7 days) in relation to flowering duration may have a substantial effect on flax’s boll development, pollen viability, pollen production, flowering, seed oil quality, quantity, and seed set. Flax fibre does not need high temperatures. Flax grows best and produces the highest-quality fibre under circumstances that are damp, overcast, and moderately chilly (18–20 °C). High temperatures, especially terminal heat, which is limiting for flax growth, cause poor adaptation of superior fibre linseed genotypes to hotter climates. Although there have not been many studies on how higher temperatures affect flax development, physiological processes, and yields [113,114], the molecular dissection is also unclear.

## 3. Biotic Stresses

Biotic stresses are responsible for major yield losses [115]; fungal infections, a few viruses, and a phytoplasma are the principal causes of flax biotic stresses. Neither bacteria nor nematodes are known to cause major stress in flax. All varieties of flax (*L. usitatissimum* L.), such assolin flax (low linolenic acid/vegetable oil flax), linseed (industrial oilseed flax), and fibre flax, are susceptible to fungi. The varieties of each species of flax may react differently to certain diseases or races of the same pathogen. In the parts of the globe where flax is grown, flax diseases vary in frequency, severity, and relevance from one location to another. The most feasible and effective method for engineering disease resistance in crop species is through breeding. Early attempts to enhance flax focused on breeding disease resilience. The morpho-physiological symptoms after biotic stresses in linseed are the yellowing of leaves, a reduced number of seeds, premature ripening (leading to yield loss), and early plant death [116]. Three types of resistance have been found according to the number of genes that influence inheritance: Resistance can be controlled by one gene (monogenic), a few genes (oligogenic), or several genes (polygenic) [117].

### 3.1. Rust Resistance

Flax-growing places around the globe have documented cases of rust. *Melampsora lini* (Ehrenb.) Desm., a fungal organism, is the causal agent of rust in flax crops. Rusts have the potential to be the most destructiveplant diseases, and flax rust is no exception [118]. Early infections and fast disease development, which are made more likely by meteorological conditions, can totally decimate flax plants and result in a significant reduction in the production and quality of both fibre and seeds. The main difficulty in producing resistant cultivars is the regular emergence of new races [119] of the pathogen. The availability of genetic resistance sources for most flax-breeding programmes globally has been credited with the disease’s rather good global management. 

Bright orange and powdery pustules that appear on leaves, stems, bolls, and other aerial plant parts are the primary indications of rust in flax. On leaves and bolls, these pustules are often round, although they are elongated on the stems. Depending on the interplay between a person’s ethnicity and genetic makeup, chlorotic or necrotic zones may develop surrounding the pustules. Severe outbreaks result in the leaves drying out and withering, severely defoliating the plants. Numerous urediniospores are produced by rust pustules, and they easily detach and travel long distances in the wind. The black telia pustules that develop from the maturing plants’ orange pustules (uredia) create hardy teliospores that can survive the winter [120].

**Figure 2:**
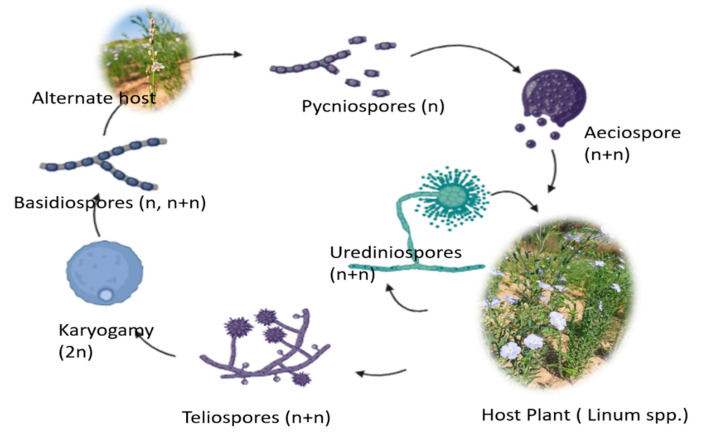
Disease cycle of rust in linseed. In this infectious stage, urediniospores are present on the main plant (host plant) and later transferred through teliospores, avoiding harsh, unfavourable conditions by remaining dormant on alternate hosts as basidiospores.

In contrast to many other rusts that need different hosts, flax rust is an autoecious rust, meaning that the fungus may go through all four phases of its life cycle—pycnia, aecia, uredia, and telia—on the flax plant (Figure 2). Field inspectors seldom ever see the pycnia and aecia, which typically occur on cotyledons and lower leaves early in the season. The stages of pycnia and aecia are crucial for the fungus to complete its sexual life cycle and for the emergence of new races. The uredia are this fungus’ third developmental stage. Since uredia develop cycles of urediniospores that might lead to new infections with each cycle during the growing season, the uredial stage is the most harmful to the crop. When the fourth stage reaches maturity, it generates tough overwintering teliospores that can endure inclement weather [120].

### 3.2. Fusarium Wilt Resistance

A typical flax biotic stress is Fusarium wilt, which is brought on by *Fusarium oxysporum*. The fungus kills plants shortly after they appear, while later infestations result in the wilting and yellowing of foliage [121]. Agrawal et al. [122] demonstrated that flax wilt resistance is acquired by recessive alleles based on F_2_ segregation ratios among nine intervarietal crosses comprising the flax wilt-resilient cultivars R552 and RLC6, along with four susceptible genotypes. Spielmeyer et al. [123] investigated the transfer of Fusarium wilt resistance in a recombinant double-haploid (DH) population produced from F_2_ seeds of a wilt-resistant (twinning Linola TM) and a wilt-prone (Australian flax cultivar Glenelg) line. The partition of two separate genes with additive effects, which accounted for 38 and 26% of the genetic diversity for Fusarium wilt resistance, was considered responsiblefor the phenotypic variance.

Spielmeyer et al. [124] used an amplified fragment length polymorphism (AFLP) marker to assign QTLs to two linkage groups. The flax varieties Mogilevsky-2, Torzhoksky 85, Kievsky, Kalininsky, Rodnik, Nika, K-65 Niva, T-17, and Ustiensky were discovered by Portyankin and Karachan [125] to be resistant to Fusarium wilt. Flax accessions from East Asia and North and South America had greater than average and mean resistance to Fusarium wilt, respectively, but flax accessions from Europe and the Indian subcontinent had lower than average resistance. This variation in resistance to Fusarium wilt was documented by Diederichsen et al. [126].

### 3.3. Alternaria Blight Resistance

Most of the world’s flax-growing regions have documented flax disorders linked to infection by *Alternaria* spp. [127]. Most reports come from India [128] and European countries where the crop is produced. The disease damages leaves and flowers as well as seedlings, producing seedling blight. In India, yield losses of up to 90% have been documented [129], and in the UK, yield losses of up to 35% have been documented [130].

According to Vloutoglou [131], the three most common species on flax are *Alternaria infectoria* Simmons, *A. linicola*, and *A. alternata*. Additional species include *A. linicola* Skolko and Groves, which is endemic to India and loves temperatures ranging from 26 to 33 degrees Celsius, as well as humidity [132]. In flax waste on and in the soil, *A. linicola* persists as thick-walled chlamydospores [131]. Flax seedlings are infected by early-season conidia. Infected seeds can spread the fungus, which results in seedling blight [131]. The seedling blight or brown rot caused by the seed-borne inoculum is the first sign of *A. linicola*. Stunted seedlings that have dark red lesions on their hypocotyls and cotyledons collapse completely in one to two weeks. A rich reservoir of inoculum is created by the diseased and dying seedlings, which spreads to infect healthy plants. On diseased leaves, dark brown lesions develop and typically combine to cover the whole leaf, which then becomes chlorotic and then dies [120].

### 3.4. Powdery Mildew Resistance

Powdery mildew, another significant flax disease caused by the fungus *Oidium lini*, was first observed in Western Canada in 1997 [133]. Ashry et al. [134] examined flax cultivars for resilience to powdery mildew. They discovered that choosing flax as a fibre variety leads to a rise in powdery mildew incidence. Choosing seed-type flax has the opposite effect, lowering resilience to powdery mildew. When Rashid and Duguid [135] investigated the genetics of resistance to powdery mildew in flax plants, they discovered that resistance to the fungus is caused by a single dominant gene (PM1). The output of flax crops is also known to be impacted by the Pasmo illness. In Western Canada, *Septoria linicola* (Speg.) Garassini has been reported to cause Pasmo disease and is common and pervasive on flax [136]. Most varieties of flax in Canada are vulnerable, and little is known about how *S. linicola* isolates are pathogenically dangerous. High-humidity and high-temperature regions show rapid illness progression. *S. linicola* thrives on agricultural leftovers that have been infected and left in the field from one season to the next.

## 4. Flax Genetic Resources

### 4.1. International

Dillman [46] underlined the importance of collecting germplasm during initial linseed breeding. Further, Zhuchenko and Rozhmina [137] described the use of landraces for fibre flax breeding. Over 48,000 flax accessions are thought to be present in ex situ collections globally, with 10,000 of them possibly being unique. The range of genetic variety maintained in the separate flax national collections (national inventory) of each unique European country, as well as the breeding techniques utilised, are essential for successful breeding activity. The European Cooperative Programme for Crop Genetic Resources (ECP/GR) network adopted the first step towards generating an inventory of the European flax gene pool at an informal meeting of flax-gathering owners within the recently built the Industrial Crops and Potato Network, among the most recent European System of Cooperative Research Networks in Agriculture (ESCORENA), held in Prague on 7 and 8 December 2001 [138].

The 2001 inventory of European flax genetic resources revealed the status. The collection of Bulgarian flax is housed in two institutions. A total 945 accessions were kept at Sadovo in 2001. Of the 450 studied accessions, 23% were linseed, 29% were flax, and 46% were of an intermediate kind. Old and landrace cultivars provided 58% of the total, followed by modern cultivars (15%) and breeder’s lines (14%). There were 12 wild species of Linum in the collection other than *L. usitatissimum*, namely, *L. austriacum*, *L. bienne*, *L. flavum*, *L. punctatum*, *L. grandiflorium*, *L. perenne*, *L. setaceum*, *L. strictum*, *L. trigynum*, *L. humile*, *L. altaicum*, and *L. viscosum* [139]. Moreover, the AgroBioInstitut (ABI) in Kostinbrod maintained 283 accessions. Outof these, 178 came from IgreSadovo, 29 were from other Bulgarian institutes, 71 were from abroad, and 5 were self-bred specimens. These accessions came from Europe, Asia, Africa, and America, respectively. The collection is made up of 54% cultivars, 27% landrace and primitive cultivars, 15% breeding lines, 1% wild forms, and 2.5% genetic stocks. By plant type, the collection was constituted by 31% fibre flax, 35% linseed, 32% combination type, and 2% other types. According to the International Union for the Protection of New Varieties of Plants [140,141], the International Flax Database (IFDB) descriptors [142,143], and other species descriptors, 259 accessions for 15 morphological traits, 5 biological characteristics, and 4 yield traits were described [144,145].

The assessment, description, and characterisation systems for flax germplasm vary depending on the needs of breeders in each country, whether it is in Europe, the USA, or Canada. To exploit the genetic diversity of flax for breeding purposes, a good, unique approach for evaluation, description, and characterisation is necessary, which should be standard at least across European nations. This is because different evaluation and characterisation techniques are employed in various European genebanks as well as outside. Thus, the IFDB may be viewed as the first input for this goal [143]. The IFDB was initially overseen and managed by Agritec Ltd. (Viale delle Terme di Caracalla, 00153 Roma (Italy)) starting in1993 in the framework of FAO ESCORENA Flax and Other Base Plants Network (FAO FOBPN) [146] and then, over time, the framework of IPGRI Coordination Group Network for Sugar, Starch and Fibre Crops (CGNSSFC), currently namedSugar, Starch, Fibre Crops & Aromatic Plants Network (CGN SSFC&APN) at Bioversity International. In theACCESS IFDB framework, it currently contains passport information for 8385 accessions of 11 collections from 10 nations. It is estimated that 37% of the accessions are unique based on a study of the provided data. It also showed that there were significant variations in the collections’ description fill rates. Table 1 displays the findings of current germplasm characterisation and assessment research carried out at the Plant Gene Resources of Canada (PGRC), Canadian National Seed Gene Bank [147].

### 4.2. National

Cultivated forms have been reported to be rich in diversity in the Indian region. A total of 2627 accessions, comprising 2495 indigenous, 51 released, and 132 foreign varieties, were preserved in long-term and medium-term storage at the National Bureau of Plant Genetic Resources (NBPGR), New Delhi, India. According to the information shown below, the Central Variety Release Committee (CVRC) and States Variety Release Committee have released and notified 51 improved varieties for general cultivation in various agro-climatic conditions. Table 2 lists the different varieties of linseed grown in different climatic regimes of India [147]. Table 3 contains list of linseed varieties that are tolerant to various abiotic and biotic stresses.

## 5. Breeding through Conventional Methods

Collections of flax and linseed genetic resources are researched for breeding purposes, and the selection of the first practical parental patterns is commonly debated. According to Tadesse et al. [148], who conducted research on crossings between high-yielding and low-yielding flax components, it may be beneficial to hybridise low- and high-yielding flax lines in order to increase genetic diversity and produce high-yielding flax lines. According to the findings of Pavelek [149], crosses of high-yielding flax components may be productive when the genetic variability of the parental lines is substantial. The largest linseed growers and producers in the world are India, the USA, and Canada. Consequently, increasing seed production, increasing oil content and quality, snowballing resilience to diseases and pests, and developing early-maturing accessions are the key breeding objectives.

Today’s breeding efforts are mostly focused on traits that stabilise yields, such as resistance to fungal infections, and qualitatively novel contents of materials. Mutagenesis [150,151], genomic approaches [152], and other methodsare used to develop new breeding techniques; for example, anther culture, mutation breeding, molecular-marker-based analysis [153], and techniques of transformation can be employed to find a solution when the genetic variability’s options have been exhausted and are no longer available. For breeding fibre flax, the most crucial factors are fibre content and resistance to Fusarium and scorch, whereas for linseed, the most crucial factors are seed production, fatty acid composition, and resistance to Fusarium and rust [154].

### 5.1. Interspecific Hybridisation

Although many interspecific crossings have been made, only those involving species with a similar number of chromosomes have so far proved beneficial. The F_1_ offspring of hybrids between *L. usitatissimum* and a few other species with n = 15 chromosomes are robust and fruitful, and they have been exploited to introduce certain desired traits, such as resistance to linseed rust.

Through modifications to the pollination of the cultivated species, interspecific hybridisation can also increase genetic variability. Some Linum species, including *L. grandiflorum*, *L. anstriacum*, and *L. perenne*, are incompatible with one another. Numerous attempts were made to cross the cultivated linseed (*L. usitatissimum*) with the self-incompatible species without success. When *L. usitatissimum* was employed as the female parent in crosses, abortive seeds that were dried up and unable to germinate were obtained [155]. The embryo of the cross between *L. usitatissimum* and *L. grandiflorum* degenerates after 7 days due to somatoplast sterility, according to embryological investigations. By cultivating the embryos of the cross and transmitting the incompatibility mechanism to the cultured species, attempts are being made to raise the hybrid. Induced self-incompatibility would force cross-pollination and raise genetic variation in farmed plants [147].

### 5.2. Hybrid Linseed

Knowledge of floral morphology and pollination methods is necessary to produce hybrid varieties with vigour or heterosis effects. Flax heterosis has ranged from 40% [156] to 231% [157]. The development of flax and linseed hybrids is less common than that of sorghum, maize, sugar beet, onions, or pear millet. The primary cause is that the male sterile flax flower has a tiny corolla that does not open to facilitate cross-pollination. Several researchers have investigated the issue of the male sterile flax, including Kumar and Singh [158,159], Dubey and Singh [160,161], and Kumar [162]. Particularly in India, several researchers have looked at the compatibility of flax or linseed kinds in diallel crosses, allowing for the assessment of the heterosis impact. Pant and Mishra [163] found positive and substantial heterosis for seed yield per plant at 40 crossings and fibre yield at 13 crosses. This impact frequently results from dominance, non-allelic interaction, or the combined influence of additive genes.

Trait selection is influenced by the relationship between the genes expressed by either the general combining ability (GCA) or the specialised combining ability (SCA). In contrast to the nonadditive effects of SCA, which were confirmed by Sood et al. [164] for number of seeds per capsule and seed weight, the GCA of flax and linseed is related to traits like the number of capsules per plant, plant height, the weight of capsules per plant, and seed yield. There were occasionally both additive and nonadditive gene effects seen [165].

### 5.3. Mutation Breeding

Despite Tammes reporting a mutation in linseed as early as 1925, nothing is known about the nature, induction, and use of induced mutations in flax. Mutagenesis in linseed has been attempted using both physical [166,167] and chemical mutagens [168,169]. Deshpande [170] identified flax seedlings with low levels of chlorophyll in the NP-12 normal group. Levan [171] discovered three families segregating for chlorophyll mutations in diploidplants but not in tetraploid plants following X-irradiation. Three different linseed varieties, Hira, Mukta, and Neelum, were exposed to radiation of10, 20, 30, and 40 kR by Rai and Das [167].

Along with various kinds of chlorophyll mutations, an increase in mutation frequency was seen with increasing irradiation dosages. The observedcholorophyll mutants showed delayed blooming, shorter plants, and fewer capsules per plant. In contrast to normal plants, these mutants had a much higher number of non-bearing tillers per plant. Sharov [172] reported mutants with good yield and a 52–56% increase in resistance to *F. oxysporum* following chemical mutagenic therapy. After irradiating the variety Neelum, George and Nayar [166] acquired a dwarf mutant (TL-1), which developed 30 days quicker than the typical plant. It is interesting to note that the mutants had larger 1000-seed weights and lighter-coloured oils with higher oil contents.

In Australia and New Zealand, three new linseed cultivars, known as “Wallaga”, “Argyle”, and “Eyre”, have been introduced under the generic name “Linola”. Golden-yellow seeds are seen in “Linola” varieties. “Eyre” is a result of a single-plant selection made in the F_4_ of the cross “Glenelg”/CPI 84495//4*”Zero”, from which an F_8_ bulk was obtained. The EMS (ethyl methane sulphonate) mutagenesis of the Australian linseed cultivar “Glenelg” and the recombination of two mutant genes produced the low-linolenic acid genotype “Zero” [173].

Eight Indian states, namely, Madhya Pradesh, Bihar, Gujarat, Delhi, Punjab, Rajasthan, Uttar Pradesh, and West Bengal, are focused on linseed breeding [174]. India’s linseed breeding industry is well established and well organised. The Indian Central Oilseeds Committee (ICOC) increased its linseed research activities in 1947, and then in 1958, the project “Intensification of Regional Research on Cotton, Oilseeds and Millets” (PIRCOM) took over. The All India Coordinated Research Project on Oilseeds (AICRPO) was launched by the Indian Council of Agricultural Research in 1968 [174] utilising international methods. 

## 6. Advanced Methods in Linseed Breeding

### 6.1. Molecular Diversity

Flax genetic diversity can be assessed using a variety of methods, including physical traits, isozymes, and molecular markers. While isozymes are scarce, morphological characteristics are frequently more quantitative and reliant on their surroundings. The prevalence and distinctiveness of DNA markers are some of their benefits. They are helpful for identifying varieties and assessing DNA diversity. To examine the genetic variation in flax, various molecular markers, such as restriction fragment length polymorphism (RFLP), random amplified polymorphic DNA (RAPD), amplified fragment length polymorphism (AFLP), and simple sequence repeat (SSR), have been developed. 

By using RAPD markers, Fu [29] evaluated the geographic trends of flax variability in the global collection of farmed flax. A total of 149 RAPD markers were measured for each of the 2727 flax accessions, which came from 63 different nations and 1 unidentified group. The accessions were divided into 12 main areas, which explained 8.2% of the RAPD variation. The most varied accessions came from East Asia and European areas, but the most distinctive accessions came from the Indian peninsula and Africa. Genetically, accessions from the West Asia area were less related to those from the Indian peninsula and more related to those from Africa. These discoveries are crucial for comprehending the domestication of flax and are also helpful for categorising the intraspecific variety of cultivated flax, identifying the fundamental subgroup of the flax collection and looking into novel gene sources for flax improvement.

Cloutier et al. [175] conducted the first comprehensive study on the production and analysis of a considerable number of SSR markers in flax. The genetic relationship among 635 genotypes was discovered by using 275 variable EST-SSRs. The pedigree connection between the accessions was associated with subclusters within the major clusters. The expressed sequence tags-signature sequence tags (EST-SSTs) created here signal the beginning of SSR markers in flax on a wide scale. They could be applied to the creation of physical and genetic maps, studies of genetic variation, association mapping, and cultivar identification.

Several molecular methods are presently available to identify germplasm at the DNA level. Due to the large number of markers that can be produced per analysis, AFLPs are typically regarded as being comparatively strong in germplasm analysis when compared to alternative genetic marker systems. In the research by Van Treuren et al. [35], duplicate germplasm in the flax collection of the Netherlands’ Center for Genetic Resources was identified using AFLPs. ANOVA was used to compare accessions in pairs in order to find duplicate genotypes. The 29 accessions of breeder’s lines could be reduced to 14 by stepwise bulking accessions until all surviving accessions were noticeably distinct. This bulking strategy only had a negligibly tiny negative impact on the among-population component of variance, demonstrating a 2.6% decrease. This outcome is addressed in connection to enhancing collection management effectiveness.

### 6.2. Association Mapping

Association mapping studies are crucial throughout animal and plant research because they aid in the discovery of genes that influence phenotypic variation and create the framework for the recognition of genes that play a vital role in what is seen as variation. Genome-wide association studies (GWASs) have become a more common practice in the quickly developing genomic age. The early accessibility of complete genome sequencing data in humans appears to have contributed to the broad application of GWASs throughout human research before it was adopted by plant science. Several plant genomes have been mapped as a result of the significant advances in sequencing technology over the past ten years. The availability of plant whole-genome sequences gave researchers the chance to perform effective GWASs [59].

Traditionally, biparental crosses, which are very simple to create, have been used to identify quantitative trait loci (QTLs) in plants. Biparental hybrids do have some restrictions. Since the community is descended from two parents, it only works with genetic variance within the parental lines, which is the most important restriction. Additionally, biparental QTL mapping has poor precision. A GWAS, in comparison, has several advantages over QTL mapping, including high resolution and the use of genetic variation present in readily available germplasm resources. The genetic makeup of the targeted material, phenotypic diversity, and the quantity and distribution of molecular markers have a significant impact on the effectiveness of GWAS.

Single-nucleotide polymorphism (SNP) markers have all the characteristics needed for high-throughput genotyping and are widely available and evenly dispersed throughout the complete genome. The majority of GWASson plants are conducted on important species like rice, maize, and wheat, as well as model species like *Arabidopsis*. Limited attempts have been made to perform GWASs on flax compared to the main food products and model species. Since flax is an oilseed grain, its breeding programme focuses mainly on increasing the seed oil content and total output. Secured output is heavily reliant on climatic factors that exert various biotic and abiotic pressures [59].

Plant resistance against stresses is governed by a sophisticated polygenetic system. GWAS is a cost-effective and effective way to analyse such intricate genomic control. For the recognition of significantly associated loci and subsequent candidate gene identification, an in-depth knowledge of the level of linkage disequilibrium (LD), the profile of LD variations across the entire genome at substantially associated loci, and functional annotations of predicted gene models is required [59].

### 6.3. Extent of Linkage Disequilibrium

The degree of LD differs significantly between various chromosomes and loci on the same chromosome, and it is not consistent across plant species. Population drift, selection, admixture, inbreeding, and taming are some of the factors that influence LD. The rate of recombination, transversion, translocation, and chromosomal duplications all play a significant role in the LD variation at various genetic locations. Numerous plant taxa have been the subject of in-depth research on LD decays. Overall, species have been found to cross-pollinate more frequently and experience very little LD degradation. For instance, it has been found that LD decays occur within 0.3–2 kb [176,177]. However, self-pollinated species like soybean have shown evidence of LD decay after extremely lengthy distances [178]. According to Sonah et al. [178], distinct LD decays at r^2^ = 0.2 were observed in soybean across various chromosomes, spanning from 250 kb to 2.5 Mb on average. Similarly, the flax genome exhibits comparatively delayed LD decays relative to cross-pollinated species since it is a self-pollinated plant species. Soto-Cerda et al. [179] used genetic information from 448 microsatellite markers and 407 flax accessions from around the world in a genome-wide analysis. According to Soto-Cerda et al. [180], LD declined across the entire genome within an average of 1.5 cM. Like the previous studies, [181] carried out two additional genome-wide investigations using 460 SSRs for 390 accessions and 112 SSRs for 407 accessions, respectively, and found LD decay up to 1.8 cM. Aside from the availability of the whole-genome sequence, insufficient attempts have been made to carry out an effective LD analysis and consequent GWAS.

### 6.4. Genetic Loci Identified by GWAS

One of the main goals of flax breeding programmes is to increase the amount of linolenic acid (LIN) in the plant. It is difficult for breeders to increase LIN content without influencing other fatty acids (FAs) because LIN content has negative correlations with linoleic acid (LIO), stearic acid (STE), palmitic acid (PAL), and oleic acid (OLE) [182]. Some flax accessions with high LIN were discovered through careful evaluation of a wide range of germplasm, but they are not well suited to cropping systems [183,184]. Identification of the genetic regions controlling FA composition is crucial in this respect. Nine loci that are strongly linked to five seed quality characteristics have been found in flax. For the iodine index, LG8 linkage group (LG), LG5 linkage group (LIN), total oil content, and LG9 linkage group (LG), the loci with the largest impacts have been described. The markers on LG2 (Lu2046) and LG6 (Lu2555), however, described about 8 and 4% of the phenotypic variance in PAL and OLE, respectively. The research was unable to find substantially linked loci for PAL and OLE. The reason that there is a negative association between LIN and other FAs may be explained by several markers associated with various FAs that were discovered to be colocalised in this research. It is interesting to note that Soto-Cerda et al. [181] found a locus linked to genes expected to be involved in FA biosynthesis.

For instance, it was discovered that the acyl-CoA: diacylglycerol acyltransferase A (*dgatA*) gene is located near the strongly linked locus on LG3. Earlier, a biparental quantitative trait locus (QTL) mapping strategy was used to identify flax *dgatA*. As a result, the loci found in flax using the GWAS method appear promising. 

### 6.5. Molecular Mapping of QTLs

Genetic mapping has been used widely in both animal and plant species since the finding of DNA-based identifiers. After Botstein et al. [185] released the initial report of restriction fragment length polymorphism (RFLP), genetic mapping efforts increased, but they were hampered by the lack of an effective study. Early in the 1990s, advances in mapping analysis led to the first genetic map spanning the complete set of chromosomes, becoming available and being reported in humans and some plant species. The first RFLP-marker-based linkage map was created in 1988 and used for QTL mapping in plants [186]. The first inheritance studies and genetic mapping of flax were conducted in 1930 by Henry, who looked at how rust protection was passed down in the Bombay and Ottawa 770B varieties. These mapping initiatives, however, were based on physical traits. The first genetic linkage map of flax was created using RFLP and RAPD markers following the development of DNA-based molecular marker methods [187]. The 1000 cM genetic linkage map created was made up of 15 unique linkage groups with ninety-four evenly spaced markers. The F_2_ mapping population created from the hybrid between CI1303 and Stormont Cirrus was used in the research. Multiple investigations have since discovered QTLs for various flax characteristics. Cloutier et al. [175] constructed a genetic linkage map utilising114 SSR markers and 5 SNPs in a DH population of 78 individuals obtained from a crossing of SP2047 (yellow-seeded withlittle linolenic acid) and UGG5-5 (brown-seeded withelevated linolenic acid). For LIO, LIN, and the iodine value (IOD), two significant QTLs were found for each, and one major QTL was found for palmitic acid. The QTL QPal.crc-LG9 explained 42% of the trait variance in palmitic acid.

In related research, Kumar et al. [188] used a recombinant inbred line (RIL) population of 243 individuals from a hybrid between cultivars CDC Bethune and Macbeth to create a genetic map using 329 SNPs and 362 SSRs. For a total of 14 distinct characteristics, 20 QTLs were found in their study. There were three QTLs for STE and OLE, two QTLs for iodine content and LIO, and two QTLs for seed protein, oil content, thousand-seed weight, cell wall, straw weight, output, seeds per boll, and days to maturity. Candidate genes for characteristics related to yield component traits, cell wall synthesis, fibre production, and fatty acid biosynthesis were discovered through the analysis of the QTL.

## 7. Integrated Omics Approach

**Figure 3:**
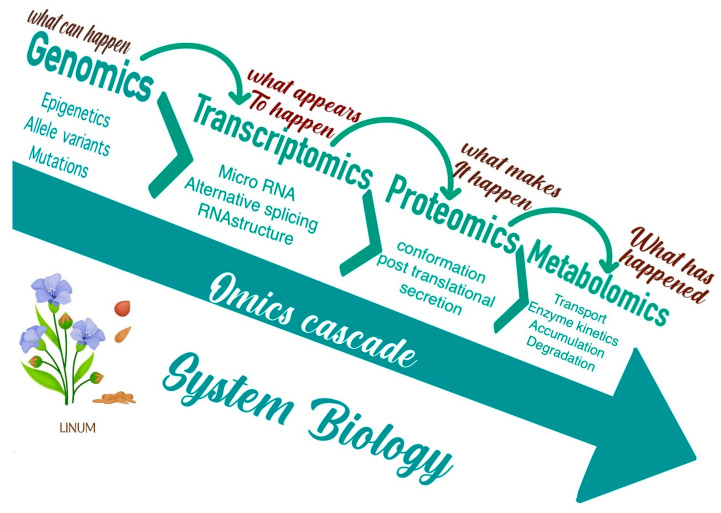
An overview of the use of omics approach, which depicts how the information is passed on from genomics to transcriptomics, then to proteomics, and finally to metabolomics, which bring about the final change in response to a stress or signal.

Among the major components of omics are transcriptomics (gene regulation and protein identification and effects, expression profiling), metabolomics (pathway and intermediates, metabolite profiling, regulation), phenomics (automated study and analysis of phenotypic and physiological effects), transcriptomics (gene regulation and expression profiling), proteomics (protein identification and effects), and ionomics. According to in-depth studies, various omics mechanisms and their combinations (Figure 3) are crucial for comprehending plant systems biology [189,190,191].

Omics-aided technologies have been utilised to conduct stress tolerance research on rice [192], soybean [193], and flax [194]. In contrast to other crops, however, comparatively fewer attempts have been made to use the available genetic and genomic tools for flax development. In accordance with Akhmetshina et al. [195] and Shivaraj et al. [59], who investigated the application of omics-assisted breeding and high-throughput sequencing technologies for the advancement of climate-smart flax, advanced tools such as genome-wide association studies (GWASs) and genomic selection in conjunction with various omic technologies offer an opportunity to increase the precision of flax improvement and plant selection.

In order to introduce weather-resilient characteristics into flax cultivars for long-term productivity, an extensive strategy incorporating a variety of technologies can significantly simplify the process [196].

### 7.1. Genomics

The genetic diversity of flax was evaluated in the early years of the twenty-first century using a variety of molecular markers [187,197,198,199,200]. The release of the flax whole-genome transcriptome provided a significant advantage in the creation of genomic tools [201]. The information from reduced representation sequencing and whole-genome re-sequencing has since been successfully applied to comprehend crop diversification, construct linkage maps, recognise markers, and discover QTLs in flax. Using the genotyping-by-sequencing (GBS) method, 258,873 SNPs spread across fifteen linseed chromosomes have been reported [188].

Recent studies have used genome-wide association studies (GWASs) to find potential SNPs for various traits, including improved stress tolerance indices, yield, and other traits in different species, including sorghum [202,203,204], rice [205], and sesame [206,207]. Although the AQP gene family improves drought resilience, multidrug and toxic compound extrusion (MATE) mediates the reaction to abiotic stresses [208]. However, research success in flax has beenless rapid than in cereals [209,210]. He et al. [211] used GBS to find 258,873 SNPs from the Canadian flax accession (core collection) as part of a GWAS to discover genetic areas linked to Pasmo resistance. Of the 500 potential QTLs found, 45 covered 85 resistance genes. Additionally, two potential genes (Lus10031043 and Lus10020016) for flax protection against this pathogen were found to be similar based on orthology with *Arabidiopsis thaliana* genes. In recent years, You et al. [212] examined 447 flax accessions for resistance to powdery mildew over 5–8 years from three sites, encompassing 372 samples from the primary collection and 75 mating lines.

**Figure 4:**
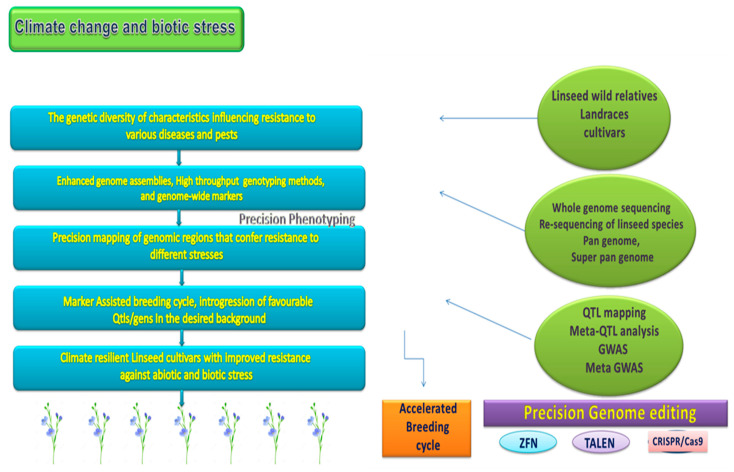
Diagrammatic illustration of the importance of genomics in the production of high-yielding linseed cultivars.

By estimating the genetic potential rather than looking for specific QTLs, genomic selection (GS) is a breeding technique that overcomes the restrictions of marker-aided selection (MAS) for pace breeding (Figure 4). GS has the capacity to resolve all variations in the genetics of complex traits, as opposed to the traditional agricultural breeding technique, which is slow in concentrating on the complicated and barely heritable quantitative variables. Because of this, it is becoming clear that using molecular genetic markers to create brand-new models based on markers for genetic assessment is a promising method [196]. The precise phenotyping of a selected diverse collection of genotypes (for the purpose of breeding) in various conditions is required in order to develop a statistical model (GS model) that can be used to calculate genomic-predicted breeding values in the breeding population. As it addresses the minor effects of QTLs, the GS technique has many benefits over traditional and marker-assisted breeding [213].

Because it combines all known molecular markers with phenotypic information, GS has been promoted as the most effective technique for determining genetic values for selection [214,215]. An enhanced genetic gain/unit period was observed during the breeding cycle in flax GS experiments [216,217]. Table 4 and Table 5 represent various QTLs/QTNs linked to different stresses in flax. For QTL mapping with GS model optimisation, they employed three biparental populations created by mating high-yielding with high- and low-ALA acid content lines. He et al. (2019) [211] developed one of the most accurate genetic prediction models for resistance to plant diseases for the genetic resilience of flax to *Septoria linicola*. You et al. [212] used 447 flax inclusions as an experimental population to create the most current model for forecasting the evaluations for powdery mildew across five years at three separate sites. The 349 QTNs discovered through GWAS described ninety-six percent of the variance in powdery mildew, demonstrating the model’s strong predictive power and promise for use in genomic prediction.

More comprehensive GS studies are anticipated soon due to the enhanced genetic techniques with genomic resources for linseed, which could help in the release of new cultivars suited to requirements. Due to greater costs than MAS, the more widespread use of GS is currently still a problem. Budhlakoti et al. [216] reviewed the present state of genomic selection research in crop plants and the prospects for its effective application in the making of weather-resilient crops. They stressed that research on genetic makeup under heat with drought stress can invariably speed up the production of stress-resilient cultivars through genomic selection [196].

### 7.2. Transcriptomics

The discovery of important genes engaged in the stress tolerance process is aided by transcriptome profiling, which offers a thorough overview of gene expression and regulation. Depending on the abundance of generated genomic materials and plant variety, different methods are used to investigate the transcriptome, including spotted microarrays, expressed sequence tags, Affymetrix Gene Chips, sequencing combined with suppression subtractive hybridisation, and RNA sequencing. The advancement of next-generation sequencing technologies and RNA sequencing has made them the most efficient, affordable, and high-throughput transcriptomic techniques [196].

Numerous transcriptomic studies have been conducted to date to determine the impact of salt and drought on oilseed products like sesame [207], flax [220], jatropha [221], sunflower [222], and soybean [223]. The sequencing of the linseed genome and the presence of genetic maps [182,195,201] set the groundwork for numerous transcriptomic studies and the discovery of genes underpinning characteristics of economic and agronomic significance. High-throughput sequencing was used to examine how flax responded to the alterations in the levels of salt and pH [224,225,226], drought [227], nutrient deficiency [228,229], and metal stress [230].

Dash et al. [227] performed a transcriptome analysis on the relatively drought-tolerant flax cultivar of India, i.e., T-397, and expression profiling assisted in identifying markers for the identification of drought-resistant flax varieties. Shivaraj et al. [231] showed the increased expression of integral membrane proteins, primarily aquaporins, using transcriptome analysis data, which improved the knowledge of their physiological function. In a transgenic strain of flax cv. Blanka, the overexpression of the *DREB2A* gene confers drought resistance [73]. Like this, only a small number of flax genes have been identified and functionally characterised under extreme temperature stress. According to Saha et al. [111], 34 potential HSF genes from the flax genome were identified across the entire genome. 

Under conditions of heat stress, different expression patterns are bestowed by HSF, along with NAC domain TFs. By lengthening roots, reducing membrane injury, and improving ion transport, two salt-tolerant genes that are related to *Arabidopsis* Senescence-Associated Gene 29 (SAG29) may improve salt tolerance. The flax transcriptome reaction to acidic soil revealed genes with altered expression patterns [232]. Through transcriptomics, the reaction of flax to an unfavourable zinc shortage and soil acidity identified genes incorporated in cell wall biogenesis photosynthesis and ion transfer [224]. Several pathogen-related dominant genes were induced to counteract the negative impacts of low Zn concentration. Melnikova et al. [228] found 96 conservative microRNA homologs from 21 families, and they also described the function of 7 microRNAs (miR408, miR395, miR168, miR398, miR169, miR399, and lus-miR-N1) in the control of metabolism and gene expression in plants during nutritional stress.

As a defence strategy, plants’ transcriptional and translational profiles are also altered by pathogen assault, which causes the implementation of various metabolic pathways and genes. In their analysis of the gene reaction during the early phases of Fusarium infection, Kostyn et al. [233] found 47 genes responsible for antioxidant biosynthesis and phenylpropanoid pathways in flax. Hundreds of genes with differential expression in reaction to early pathogenesis were found in the prevalent Canadian cv. CDC Bethune transcriptome of flax immune to Fusarium wilt and a susceptible variety, Lutea. Several important genes, including those involved in secondary metabolism, the stimulation of pathogenesis-related (PR) interactions, and the formation of lignin, have higher transcript abundance among these [196]. The transcriptome of two susceptible and two resistant BC_2_F_5_ populations, as well as four cultivars of fibrous flax, revealed the enhanced expression of many genes involved in the defence response, such as PR protein-encoding genes, ROS production genes, and genes related to cell wall formation [234]. 

Boba et al. [235] recently found that upon Fusarium infection in flax, upregulation of the terpenoid pathway, resulting in higher ABA content, initiates the fast plant response, and PR genes, particularly chitinase and 1,3-glucanase, play an important role in resistance. In previous research, Wróbel-Kwiatkowska et al. [236] found that transgenic flax plants overexpressing the 1,3-glucanase gene displayed reduced vulnerability to this pathogen. In reaction to infection by *Fusarium oxysporumlini*, it was discovered that the resistant flax cultivar’s transcriptomic response was faster and more effective, enabling the translation ofa greater number of activated and repressed genes [237]. 

The NCBI Sequence Read Archive and NCBI Gene Expression Omnibus libraries contain the transcriptome sequences, and gene expression data for flax. *L. usitatissimum* microRNA sequences are provided in the “miRbase” database, along with their main and secondary structures and locations within the flax genome. In the field of flax transcriptomics, there are more studies published (Table 6) on tolerance to abiotic stressors than on resistance to biotic stresses, which is significant and might be a result of targeted characteristics under breeding programmes for areas. Most of the transcriptomic research only looked at one or two genotypes; however, for comparative analysis and gene annotation, a larger number of varied genotypes should be examined [196].

### 7.3. Metabolomics

Interpreting the mechanisms of plant biology requires integrating metabolomics because metabolites, which are the consequences of gene expression, provide a detailed picture of an organism’s biological and physiological condition [243,244,245]. However, as discussed by Hall et al. [246], metabolomic research is not without its difficulties because the extent of the plant metabolome is unclear. Plants have a widervariety of metabolites than other species. A wide range of statistical channels, involving mass spectrometry (MS)- and nuclear magnetic resonance (NMR)-based techniques like CE-MS (capillary electrophoresis–mass spectrometry), GC-MS (Gas Chromatography–Mass Spectrometry), FTIR, and LC-MS (Liquid Chromatography–Mass Spectrometry), have been used to identify and quantify awide variety of primary and secondary metabolites produced in response to abiotic and biotic stresses. NMR is usually used to identify moderate-to-high-abundance metabolites, and it requires little sample preparation.

Additionally, new improvements in NMR superconducting magnet field intensity have led to better spectral precision and detection sensitivity. In contrast to NMR, modern MS-based methods produce analyses of complicated plant metabolite mixtures with greater sensitivity. A review paper by Ibáez et al. [247] provides a summary of the most contemporary literature on direct ionisation methods used in the metabolomics of food. It is well known that metabolites like lignans, particularly omega-3 fatty acids and polyunsaturated fatty acids (PUFA), are likely to induce a positive impact on nourishment and the avoidance of some diseases [248,249]. In order to choose flaxseed types with a superior nutrient composition, a tool based on NMR metabolomics was developed [250]. Lignans and omega-3 fatty acids, two bioactive substances of importance for human health, are significant components of flaxseed. Oilseeds have been found to accumulate a wide range of secondary metabolites, such as sugars, inorganic ions, glycine betaine, and proline, in addition to the metabolomics of nutritive compounds, to promote plant adaptation to abiotic stress [251].

BABA, for example, has been shown to play a part in promoting drought resistance in a variety of plants, including *Arabidopsis* [252], apple [253], rice [254], spring wheat [255], potato [256], and tomato [257]. More osmoprotectants, anthocyanins, and proline were accumulated when PR1, PR2, and PR5 genes were overexpressed in *Arabidopsis*, while trehalose production made tobacco more drought-tolerant [258,259]. In reaction to water stress, BABA in flax increases proline and nonstructural carbs while decreasing aspartate concentration and inorganic solutes [260]. Flax [261], along with rice, was shown to have relatively higher concentrations of proline and glycinebetaine under salinity stress [262].

**Figure 5:**
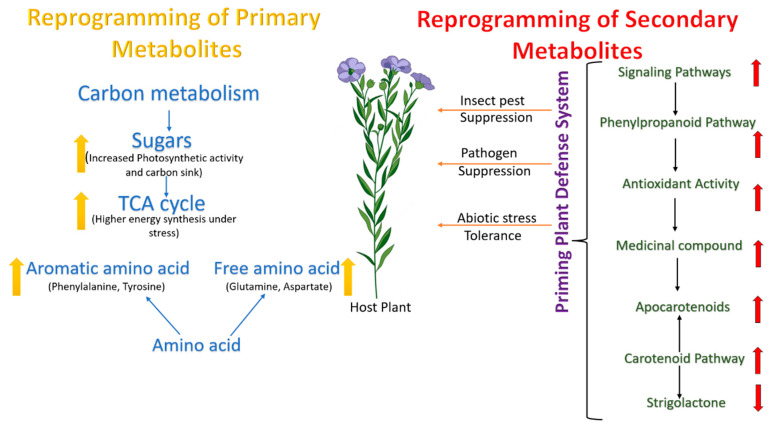
Schematic representation of pathways that are up- and downregulated on encounter with various types of stress and respond by changing metabolite concentrations.

Total soluble sugars, compatible solutes, and total protein contents, like proline and betaine, were found to increase with increasing salinity in flax genotypes under PEG-induced water stress and saline–alkaline environments, suggesting that they may play a role in adjusting to osmotic stress [263]. Under salt and/or osmotic stress, different levels of both wild-type and PLR-RNAi transgenic flax, lipid peroxidation, and a distinct metabolic profile of MDA have been noted [261,264]. As a defence strategy, pathogen assault also causes changes in several secondary metabolites, including lignin, flavonoids, polyamines, catecholamines, terpenoids, tannins, phenyl propanoic acids, and phenolics, which are produced as a result of the translational profile of plants. Many rice studies related to metabolomics have been conducted to identify important metabolites and routes in reaction to different types of environmental distress [265]. The purpose of these studies was to comprehend how the model crop rice induced defence mechanisms involving effector-prompted immunity (ETI) and PAMP-triggered immunity (PTI) in response to pathogens. As a result, stress causes significant alterations in a plant’s metabolic makeup (Figure 5), and full metabolite profiling may offer important insights into processes for stress tolerance [196].

### 7.4. Proteomics

Proteomics is the real-time analysis of the molecular and functional properties of every protein in a living thing. It combines different bioinformatic techniques with two-dimensional (2-D) gel electrophoresis, Western blotting, mass spectrometry (MS), matrix-assisted laser desorption ionisation–time of flight (MALDI TOF), and ELISA [266,267]. Recent advances in proteomics have decreased protein evaluation mistakes and opened new avenues for high-throughput proteome studies. Most of the proteomic research has been undertaken on the entire genomes of soybean, wheat, barley, rice, and potatoes. Reports on proteomic research on oilseeds include those on flax [268,269], sunflower [270], and Indian mustard [271].

Proteome analyses have shown that tolerant plants have consistently increased levels of stress-reactive chains of amino acids compared to their sensitive counterparts, which include transcriptional regulators like Myb protein, B-Peru-like protein, and SWIB/MDM2 protein involved in anthocyanin biosynthesis [272,273]. In comparison to their sensitive counterparts, drought-tolerant wheat and barley types were found to have higher levels of proteins, such as several chaperons (HSP70, HSP90, and cyclophilin A), glutathione-S-transferase (GST), and lipoxygenase (LOX) [274]. Another study found that drought [275], low temperature, and salt [276] caused reductions in RubisCo, enzymes like phosphoglycerokinase (PGK), and transketolase phosphoribulokinase (PRK) in wheat [277]. An increase in numerous minor heat shock proteins, as well as HSP82 from the HSP90 family, was found inside the endosperm of growing wheat seeds exposed to a heat phase [278].

Proteomic investigation and physiological responses in two drought-resistant maize cultivars demonstrated that HSP plays a substantial role in protecting plants from drought stress in a manner [279]. Recently, Halder et al. [280] summarised the proteomic research on salinity, drought stress tolerance, and root system architecture carried out in the previous ten years. They also examined the function of proteomics for abiotic stress tolerance in wheat. It is useful to characterise the proteome of tissues with pathogen infections using proteomic studies of biotic stress. Numerous potent metabolites that are responsible for resilience have been identified in a thorough analysis of the worldwide proteomics studies looking into the reactions of rice to biotic stress [265]. In flax, this area has recently begun to be studied [269]. Some of the examples are given in the Table 7.

### 7.5. Ionomics

Ionomics is the study of the elemental composition of non-metals, metals, and metalloids in various plant species, with a focus on high-throughput detection and quantification [196]. Different plant varieties’ ionomic compositions may be determined using high-throughput methods like mass spectrometry with inductive and inductively coupled plasma–atomic emission spectroscopy. It plays a crucial part in helping us comprehend the various elements, their chemical and physical makeup, and how they affect plant metabolism, nourishment, and biochemistry. Due to the diversity of soil types and various edaphic variables affecting growth, plants have developed a range of element uptake skills [286]. Additionally, transit, environmental stress, root uptake capacity, and element abundance all have an impact on the ionomic makeup of a crop. 

The quantity of accumulated Na+ and metabolites implicated in glycolysis and the tricarboxylic acid (TCA) cycle were significantly negatively correlated, according to the ionome of wild and cultivated barley that was exposed to various degrees of salt tolerance [287]. Studies on wheat [288], as well as other grasses like *Aneurolepidium chinense* [289], *Setaria viridis* [290], and flax, revealed that the concentration of Na^+^ rises with increasing alkalinity stress because plants store significant amounts of Na^+^ in their vacuoles to lower cell water potential. K^+^, Na^+^, Ca^2+^, and Cl were the major inorganic ions engaged in osmotic adjustment under PEG-induced water stress in flax, improving drought resilience [291]. In the caseof flax, there were no appreciable variations between the impacts of salt and alkalinity stress on the concentrations of Na^+^ and K^+^ in shoots [292]. This indicates that, unlike other plants like wheat and *Chloris virgata*, where the K^+^ concentration of shoots was found to be reduced under alkaline stress, flax shoots may have a different adaptive mechanism to alkaline stress. Another study on flax revealed that under salt stress, the amounts of NO_3_ in flax shoots dropped, while the uptake of Cl and H_2_PO_4_ increased significantly. Therefore, knowing the elemental makeup can help with understanding the process of stress endurance. Ionomics research in flax has not received much notice [196].

### 7.6. Phenomics

High-throughput analysis is used to research phenomics, which is the group of phenotypes combining GxPxE relationships under environmental circumstances [190]. As a result, the phenotype offers the strongest link between surroundings and plant genetics. While phenotypic characterisation has advanced more slowly over the past ten years due to improvements in sequencing technologies, this has limited the discovery of quantitative characteristics, especially those linked to stress resistance [293].

Phenotyping in reaction to abiotic stress remains a significant task because of the complex biosynthetic processes that handle the response of plants to external stressors [294]. Because genomic approaches like GWAS, GS, and QTL rely on high-throughput phenotyping for the improvement of specific traits, the significance of accurate phenotyping has increased in the postgenomic age [295]. The most promise exists for plant breeding when phenomics is used in conjunction with other omic methods. As a result, non-invasive technologies such as magnetic resonance imaging (MRI), lidar (which includes RBG digital imaging) to evaluate growth parameters, and colour imaging of biomass have all been researched to determine the elevated canopy and concealed half (root system) of plants [296,297].

Examples include employing ground-filled rhizoboxes to analyse the architecture of wheat roots using RGB imaging [298] and using RGB digital imaging to phenotype plant shoots [299]. Infrared thermography to confirm the function of stomatal electrical conductance in barley and wheat saplings under salt stress is another illustration [299]. Nuclear magnetic resonance (NMR), computed tomography (CT), and X-rays have all been employed forin situ 3D imaging of root anatomy. There are not many web databases that can help people with image processing, like http://www.plant-image-analysis.org, accessed on 5 May 2023. In order to obtain real-time phenome responses to diseases, the exterior environment, and nutrients, contemporary instruments include hyperspectral imaging systems, fluorescence imaging systems, high-throughput advanced plant phenotyping platforms, laser scanners, and high-resolution IR/NIR cameras. However, to extract phenome data from the enormous databases produced by phenotyping, deep learning techniques are required. Additionally, the thorough administration of platforms and software presents significant difficulties, restricting this application to a few key commodities, like rice, maize, and wheat [196].

## 8. Transgenic Flax/Linseed

The initial genetic modification made to flax washerbicide tolerance. The herbicide glyphosate (Roundup) blocks the enzyme 5-enolpyruvylshikimate-3-phosphate (EPSP) polymerase in higher plants. To create transgenic flax lines resistant to 5 mM glyphosate, the glyphosate-insensitive EPSP enzyme from *Petunia hybrida* was transformed into flax by an *Agrobacterium*-mediated method of transformation [300]. Later, flax was given additional herbicide-resistant genes, including those for glufosinate ammonium [301] and chlorsulfuron [302]. Under the direction of the CaMV 35S promoter, the phosphinothricin acetyltransferase (PAT) gene from *Streptomyces viridochromo* was overexpressed. The PAT enzyme was introduced into flax plants to provide them with resistance to the herbicide glufosinate ammonium [301].

To tolerate abiotic stress conditions, several crop plants have undergone genetic modification. Numerous candidate genes, including functional and regulatory genes, have been modified [302]. Functional genes, such as those that encode water channel proteins, ion transporters, late embryogenesis abundant (LEA) protein, and heat shock protein (HSP), are directly engaged in the defence of cells against stressors. The regulatory genes control signal transmission and gene expression during stress reactions, and they comprise transcription factors, protein phosphatases, and protein kinases [303,304]. These studies aiming to genetically modify flax for resistance/tolerance to abiotic stress have not yet been published. When plants are subjected to abiotic stress, the activity of the transporters is first impacted, which hinders the plants’ physiological function. The transport of water and other minor solutes, including urea, ammonia, glycerol, silicic acid, boric acid, CO_2_, and H_2_O_2_, is significantly aided by aquaporins in plants [305]. Abiotic stress resistance in various plant species has recently been effectively demonstrated by the application of genes expressing aquaporins. Aquaporins have been discovered in a variety of agricultural plants, including flax, due to their significance [306,307]. A total of 51 AQPs from various subfamilies were found in the flax genome [210].

## 9. Conclusions

The production of oilseed crops is substantially affected byan array of abiotic and biotic stressors. To better understand the chemical complexes and biochemical mechanisms involved in plants’ ability to tolerate stress, large amounts of data are quickly being created and annotated on a global scale. The availability of various genomic resources in flax has dramatically grown over the past ten years. These resources include molecular markers, linkage maps, transcriptomes, and whole-genome sequencing. Through a varietal development programme, these resources may be effectively used to increase the capacity of flax for adaptation to the environment and resistance to biotic stress. Given its great economic worth, flax is used in a variety of ways in the food, bioenergy, nutrition, and nutraceutical industries. This review has covered many aspects of tolerance that are now being utilised to overcome these barriers. These strategies really work to increase a plant’s natural ability to withstand diverse biotic and abiotic stresses, possibly having long-lasting impacts and, in theory, not negatively affecting other intriguing agricultural attributes like yield and the quality of edible parts. Additionally, technological advancements like the application of high-throughput platforms to measure the tolerance traits of crop genotypes upon treatments give researchers the chance to characterise the plant responses of a subset of varieties under stringent growing circumstances or in the field, where real-world agricultural conditions occur.

## Figures and Tables

**Table 1:** Taxa of *Linum* conserved in Canada’s plant genetic resources.

Species	No. of Accessions	Life Form	2n	Origin
*L. campanulatum* L.	1	Perennial	28	West Mediterranean
*L. austriacum* L.	3	Perennial	18	Asia, Siberia, Central and East Europe
*L. bienne* Mill.	11	Winter-annual	30	Mediterranean, West Europe, West Asia
*L. decumbens* Desf.	1	Annual	30	South Europe
*L. capitatum*	1	Perennial	34	Balkan
*L. altaicum* Ledeb.	2	Perennial	18	West Siberia
*L. leonii*	1	Perennial	18	France, Germany
*L. flavum* L.	4	Perennial	28 (30)	Caucasus, South and Central Europe
*L. trigynum* L.	2	Annual	20	Mediterranean, South and Central Europe, West Asia
*L. hirsutum* L.	1	Perennial	16	Central and East Europe, West Asia
*L. narbonense* L.	3	Perennial	18 (20)	Mediterranean
*L. rigidum* Pursh	1	Perennial	20	North America
*L. lewisii* Pursh	11	Perennial	18	North America
*L. pallescens* Bunge	1	Perennial	30	West Siberia
*L. tenuifolium* L.	1	Perennial	16	Europe, West Asia, Mediterranean,
*L. perenne* L.	10	Perennial	18	Siberia, West Asia, Central and East Europe
*L. strictum* L.	3	Annual	18 (30, 22)	Mediterranean, West Asia, South Europe,
*L. grandiflorum* Desf.	7	Annual	16	Algeria

**Table 2:** List of varieties grown under different climate regimes in India.

Varietal Characters	Name of Variety
Seed type	JLS95, JLS66, LSL93, RLC 148, Pratap 2, T 397, JLS27, PKV-NL 260, Kota Barani Alsi 4, JL41, JLS67, JLS73, Arpita, Padmini, Divya, Priyam, Indu (LCK 1108), Rajan, Surya, TL 99, Kota Barani Alsi 5, Kota Barani Alsi 6, Jawahar Linseed 165, RLC 164, RLC 167, RLC 171, Sabour Tisi-2, Sabour Tisi-3, Birsa Tisi-1, LCK 1611, JLS122, SHA5
Irrigated conditions	Pratap 2, JLS27, Kota Alsi 6, Priyam, Indu (LCK 1108), Rajan, Surya, TL 99, SHA5, LCK 1611, JLS95, JLS66
Rainfed condition	JLS95, JLS66, LSL93, RLC 148, T 397, Sharda, Padmini, KV-NL 260, Kota Barani Alsi 4, JL 41, JLS67, JLS73, Arpita, Padmini, Divya, JLS122
Utera situation	RLC 143, RLC 153, Sabour Tisi-2, Sabour Tisi-3, R 552
Cultivars with dual purpose (seed + fibre)	Him Alsi-2, Nagarkot, Jeevan, RLU-6, Rashmi, Parvati, Meera, Shikha, Tiara (JRF 2)

**Table 3:** List of linseed varieties that are tolerant to various abiotic and biotic stresses.

S. No.	Name of Variety	Year of Release and Notification No.	Pedigree	Originating Centre	Duration	Avg. Yield kg/ha	Oil Content (%)	Recommended States	Special Features
1	K-2	1975 44 E, 21 August 1975	Rust-resistant strain from UP x Kangra local	Ludhiana (Punjab)	170–175	1110(I)	40.04	Punjab, Haryana, and Uttar Pradesh	White flower, dark brown, resistant to wilt and rust
2	LC-185	1975 44 E, 21 August 1975	NP(RR)-37x Kangra local	Ludhiana (Punjab)	150–160	500(U)	38.89	Punjab	Blue flower, resistant to powdery mildew and rust
3	HIRA	1978 429 E, 19 December 1978	H 342 x NP(RR)-9	Kanpur (U.P.)	130–135	1200(R)	36.36	Bundelkhand area and U.P.	White flower, resistant to wilt and rust
4	MUKTA	1978 429 E, 19 December 1978	H 626 x NP(RR)-9	Kanpur (U.P.)	127–132	1200(I)	41.40	Eastern U.P.	Large white flower, medium- and brown-seeded, resistant to wilt and rust
5	CHAMBAL	1978 13 E, 19 December 1978	Local x RR45	Kota Rajasthan	125–130	900(I)	40.11	Rajasthan	Blue flower, brown- and bold-seeded, moderately resistant to rust, wilt, and powdery mildew
6	NEELUM	1978 13 E, 19 December 1978	T-1 x NP(RR)-9	Kanpur (U.P.)	140–145	1500(I)	43.00	Mid Central and Western Uttar Pradesh	Blue flower, brown- and bold-seeded, tolerant to wilt and rust
7	JAWAHAR-1	1982 19 E, 14 January 1982	Selection of KP 29	Jabalpur (M.P.)	116–120	900(R)	38.34	Madhya Pradesh	Red-violet flower, dark-brown- and bold-seeded, resistant to rust
8	JAWAHAR-7 (JLS-1)	1982 19 E, 14 January 1982	Selection of No. 55	Raipur (C.G.)	116–120	700(R), 300(U)	37.79	Madhya Pradesh	Red-violet flower, dark-brown and bold-seeded, resistant to rust
9	JAWAHAR-17 (JLS-7)	1982 19 E, 14 January 1982	Selection of No. 55	Raipur (C.G.)	117–120	1300(I), 800(R)	37.61	Madhya Pradesh	Red-violet flower, dark-brown- and bold-seeded, resistant to rust
10	NEELA	1982 19 E, 14 January 1982	SPS from indigenous collection	Behrampore (W.B.)	127–135	850(R)	34.93	West Bengal	Blue flower, brown- and medium-seeded, resistant to bud fly
11	LC-54	1982 19 E, 14 January 1982	K2 x Kangra local	Ludhiana (Punjab)	155–165	1330(I)	42.00	Punjab, H.P. and Haryana	White flower, brown- and medium-seeded resistant to wilt and rust
12	C-429	1983 429 E, 3 January 1983	No.3 x IP-135	Nagpur (M.S.)	125–130	1000(R)	39.07	Maharashtra	Red-violet flower, dark-brown- andsmall-seeded, resistant to rust
13	T-397	1984 596 E, 13 August 1984	T-491 x T-1103-1	Kanpur (U.P.)	120–125	1100(I)	44.00	Bundelkhand of U.P., Bihar, Assam, M.P. and Rajasthan	Violet flower, dark-brown-seeded, tolerant to rust, wilt, and drought
14	JAWAHAR-552 (R-552)	1984 596 E, 13 August 1984	Sel. Number 55x B-67	Raipur (C.G.)	118–125	900(R)	44.00	Madhya Pradesh	Violet flower, light-brown-seeded, medium toleranceto rust, wilt, and powdery mildew
15	PUSA-2	1985 295 E, 9 April 1985	Selection of BS 12	New Delhi	125–150	730(R)	38.31	Punjab, H.P., Haryana and Rajasthan	White flower, brown-seeded, medium height, resistant to rust
16	PUSA-3	1985 295 E, 9 April 1985	K2 x T-603	New Delhi	125–150	800(I)	37.65	Punjab, H.P., Haryana and Rajasthan	White flower, brown-seeded, medium height, resistant to rust
17	S-36	-	Selection of Local Solapur	Pune (MH)	130–135	400(R)	34.80	Karnataka	Blue flower, light-brown- and small-seeded
18	HIMALINI	1985 295 E, 9 April 1985	K2 x Kangra local	Palampur (H.P.)	150–175	1310(I)	42.00	Punjab, H.P., Haryana and adjoining of Rajasthan	White flower, brown-seeded, medium height, resistant to rust and wilt
19	JAWAHAR-23	1985 540 E, 24 July 1985	EC 9832 x Hira	Jabalpur (MP)	120–130	1000(I)	43.00	M.P., Odisha, Raj. Bundelkhand of U.P., M.S., Karnataka	White flower, brown- andmedium-seeded, resistant to wilt and rust and powdery mildew
20	GARIMA	1985 540 E, 24 July 1985	T-126 x Neelum	Kanpur (UP)	120–130	1490(I)	42.00	U.P. (Excl. Bundelkhand), Bihar, W.B. and Assam	Blue flower, medium height, brown-seeded, tolerant to PM, altenararia blight, and wilt, resistant to rust
21	SWETA	1985 540 E, 24 July 1985	Mukta x T-1206	Kanpur (UP)	130–135	880(R)	44.00	U.P. (Excl. Bundelkhand), Bihar, W.B. and Assam	Blue flower, mediumheight, brown-seeded, tolerant to PM, altenararia blight, and wilt, resistant to rust
22	SHUBHRA	1985 540 E, 24 July 1985	Mukta x K 2	Kanpur (UP)	130–135	1390(I), 870 (R)	45.00	U.P. (Excl. Bundelkhand), Bihar, W.B and Assam	Dark-brown-seeded, medium, white flower, resistant to rust
23	LAXMI-27	1987 165 E, 6 March 1987	Neelum/R1//Neelum x NPRR-9// Neelum x R1// Neelum x Afg-8	Mauranipur (UP)	110–120	1260(I),1020(R)	45.00	Bundelkhand of U.P.	Red-violet flower, dark-brown-seeded medium height, resistant to rust, wilt, and PM
24	GAURAV	1987 834 E, 18 September 1987	Sel.3 x EC-1552	Kanpur (UP)	137–140	1050 (S) 950(F)	43.00	Assam, U.P. (Excl. Bundelkhand), Bihar, West Bengal	Violet-red flower, brown- and medium-seeded, resistant to wilt and rust
25	KIRAN	1988 10 E, 1 January 1988	(R1x Afg-8) x R1	Raipur (C.G.)	120–126	750(R)	43.00	M.P., Odisha, Bundelkhand of U.P., M.S., Karnataka, and Rajasthan	Violet-red flower, brown- and medium-seeded, resistant to wilt, powdery mildew, and rust
26	JANKI	1988	New River x LC 216	Palampur (H.P.)	165–170	1200(I)	42.00	Himachal Pradesh	Violet-red flower, brown- and medium-seeded, resistant to wilt, powdery mildew, and rust
27	JEEVAN	1988 10 E, 1 January 1988	Summit x LC-216	Palampur (H.P.)	175–180	1090(S 1100(F)	45.00	Punjab and Himachal Pradesh	Violet-red flower, brown- and medium-seeded, resistant to wilt, powdery mildew, and rust
28	SURABHI	1995 408 E, 4 May 1995	LC 216 x LC 185	Palampur (H.P.)	165–170	1000(U)	44.00	Himachal Pradesh	White flower, yellow- and small-seeded, resistant to wilt, powdery mildew, and rust
29	NAGARKOT	1995 400 E, 4 May 1995	New river x LC 216	Palampur (H.P.)	165–170	1150 (S) 950 (F)	43.00	Panjab, U.P., H.P., W.B., Assam, Haryana and Raj.	Blue flower, brown- and medium-seeded, resistant to wilt, powdery mildew, and rust
30	SHIKHA	1997 401 E, 15 May 1998	Hira xCrista	Kanpur (U.P.)	135–140	1233 (S) 1033 (F)	42.00	U.P. (Excl. Bundelkhand), Bihar, W.B. and Assam	Blue flower, brown- and medium-seeded, resistant to wilt and rust
31	LC 2023	1998	Flake x LC 54	Ludhiana (Punjab)	158–163	1294 (I)	38.8	Punjab	-
32	PADMINI	1998 1050 E, 26 October 1999	(EC 41,628 x EC 77959) x (DPL 20 x Neelum)	Manranipur (U.P.)	120–125	943 (R)	43.00	M.P., Rajasthan, Bundelkhand of U.P., Maharashtra, Odissa and Karnataka	Blue flower, brown-seeded, resistant to powdery mildew
33	JAWAHAR LINSEED SAGAR-9 (JLS 9)	1998 425 E, 8 June 1999	(RL-102 x R7) x J 23	Sagar (M.P.)	115–125	1250 (I), 1000 (R)	42.00	Madhya Pradesh	White flower, mediumheight, brown- and bold-seeded, resistant to wilt, powdery mildew, and rust
34	RASHMI	1999 1050 E, 26 October 1999	Gaurav x Janki	Kanpur (U.P.)	135–140	1003 (S), 719 (F)	41.00	U.P., Bihar, W.B. and Rajasthan	Very light blue flower, brown-seeded, resistant to wilt, powdery mildew, and rust
35	MEERA	2000 340 E, 3 April 2000	(RL75-6-2 x RL 29-8) x LCK 8528	Kota (Raj.)	135–140	1439 (S), 1011 (F)	42.00	U.P., Bihar, W.B. and Rajasthan	Violet-blue flower, brown-seeded, resistant to wilt, powdery mildew, and rust
36	RL-914	2000 973 E, 4 September 2002	(RR 9 x R-93) x (Flake 1 x LC 54)	Kota (Raj.)	135–140	1617(I)	41.10	Kota command area of Rajasthan	Violet-blue flower, medium height, brown-seeded, resistant to wilt, powdery mildew, and rust
37	PARVATI	2001 92 E, 2 February 2001	EC 41628/ EC77959//DPL20 x Neelam///EC216 x Hira//BR 1x NP 440	Manranipur (U.P.)	140–146	1600 (S), 1020 (F)	42.00	U.P., Bihar, W.B. and Rajasthan	Blue flower, medium height, brown-seeded, resistant to powdery mildew and rust
38	SHEELA	2001 92 E, 2 February 2001	Gaurav x Janki	Kanpur (U.P.)	155–160	1379 (R)	41.00	Himachal Pradesh, Panjab, Haryana, and Jammu and Kashmir	Blue flower, shining-brown-seeded, resistant to wilt, powdery mildew, and rust
39	SHEKHAR	2001 92 E, 2 February 2001	Laxmi 27 x EC 1387	Kanpur (U.P.)	135–140	1555(I), 920 (R)	43.00	U.P. (Excl. Bundelkhand), Bihar, W.B. and Assam	Violet-blue flower, shining-brown-seeded, moderately resistant to bud fly, alternaria blight, powdery mildew, and rust
40	NL-97	2001 92 E, 2 February 2001	R-7 x RLC 4	Nagpur (M.H.)	115–120	641 (R)	42.00	Bidarbha Region	Blue flower, brown- and medium-seeded
41	JAWAHAR LINSEED SAGAR-27 (SUYOG)	2002 161 E, 4 February 2004	(Kran x KL-168) x Kiran	Sagar (M.P.)	118–125	1509 (I)	41.43	Raj. Bundelkhand of U.P., M.P., C.G., M.H., Odissa, A.P., and Karnataka	White flower, light-brown-seeded, resistant to rust, powdery mildew, and bud fly
42	BINWA (KL-210)	2004 122 E, 2 February 2005	Flake 1 x SPS-47/7-10-3	Palampur (H.P.)	179–186	858 (I)	40.00	Haryana, Panjab, Himachal Pradesh, and J&K	Blue flower, yellow-seeded, resistant to wilt and rust
43	BANER (KL-224)	2005 1177 E, 25 August 2005	EC 21741 x LC 216	Palampur (H.P.)	171–203	511 (U)	39.70	Haryana, Panjab, Himachal Pradesh, and J&K	Purple flower, brown-coloured seeds, resistant to rust
44	INDIRA ALSI-32 (RLC-76)	2005 1566 E, 5 November 2005	Kiran x RLC 29	Raipur (C.G.)	110–115	780(R)	39.18	CG, Maharashtra, Karnataka, and Odisha	Blue flower, dark-brown-seeded, powdery mildew
45	KARTIKA (RLC-76)	2005 1566 E, 5 November 2005	Kiran x LCK-88062	Raipur (C.G.)	103–108	1078(R)	42.93	Rainfed areas of Chhattisgarh	Blue flower, light-brown and medium-seeded, resistant to bud fly and powdery mildew
46	DEEPIKA (RLC 78)	2006 1178 E, 20 July 2007	Kiran x Ayogi	Raipur (C.G.)	110–115	1272 (SI&U)	41.39	Partially irrigated as well as *Utera* Situation of CG	Blue flower, brown-seeded, resistant to powdery mildew
47	SHARDA (LMS-4-27)	2006 1572 E, 20 September 2006	(Shubhra x J1) x (J1 x Kiran)	Manranipur (C.G.)	100–105	762 (R)	41.32	CG, Maharashtra, Karnataka, AP and Odissa	White flower, brown-seeded, MR to wilt, powdery mildew, and bud fly
48	PRATAP ALSI-1 (RLU-6)	2007 1703 E, 5October 2007	Acc. 750 x RL 29-8	Kota (Raj.)	129–135	1997 (S) 834 (F)	41.08	Rajasthan Kota command area	White flower, brown-seeded, MR to rust, wilt, and bud fly
49	LC-2063	2007 1108 E, 8 May 2008	1509 x LC 54	Ludhiana (Punjab)	115–125	1200 (I)	37.40	Irrigated area of Punjab State	Blue flower, dark-brown-seeded, MR to bud fly
50	HIMANI KL-214	2008 2458 E, 16 October 2008	DPL 20 x KLS-1	Palampur (H.P.)	177–200	583 (*U*)	36.40	HP, PB, Haryana and J&K	Blue flower, small- andbrown-seeded, MR to powdery mildew and rust
51	AZAD ALSI-1 (LMS 9-2K)	2008 2458 E, 16 October 2008	RL 904 x Kiran	Manranipur (U.P.)	125–130	1610 (I)	39.92	BKD area of UP, MP and Rajasthan	Red violet, MR to wilt, PM blue flower, dark-brown-seeded, resistant to bud fly and rust
52	RLC 92	2008 2458 E, 16 October 2008	Jeevan x LCK 9209	Raipur (C.G.)	111	1196 (I)	37.70	CG, Maharashtra, Karnataka, AP, and Odissa	Tinyblue flower, light-violet flower, brown- and medium-seeded, moderately resistant to powdery mildew, rust, and bud fly
53	SHIVAL (SLS-67)	2010 733 E, 1 April 2010	LCK 9610 x LMS 127	Sagar (M.P.)	108–110	1252 (R)	40.16	BKD area of UP, MP, and Rajasthan	White flower, light-brown-seeded, MR to powdery mildew and rust
54	JAWAHAR LINSEED-41	2011 2326 E, 10 October2011	Kiran x Acc. No.443	Hoshangabad (M.P.)	115–120	1600 (I)	40.00	Area of MP state with limited facility	White flower, brown- and bold-seeded, resistant to rust, powdery mildew, and rust
55	BHAGSU (KL-215)	2010 2137 E, 31 August 2010	RL-50-3 x Surbhi	Palampur (H.P.)	175–201	428(*U*)	36.38	Himachal Pradesh, J&K, Uttaranchal, Punjab, and Haryana	Blue flower, brown- and small-seeded, MR to rust
56	RUCHI (LCK 5021)	2011 283 E, 7 February 2011	Garima x LCK 88062	Kanpur (U.P.)	134	1366 (S), 1055 (F)	39.84	UP. (Excl. Bundelkhand), Bihar, WB and Assam	Blue flower, shining-brown-seeded, MR to rust, powdery mildew, and bud fly
57	JAWAHAR LINSEED SAGAR-73 (JLS 73)	2011 632 E, 25 March 2011	Padmini x Laxmi 27	Sagar (M.P.)	111–114	1090 (R)	38.82	Bundelkh and region of UP, MP, Rajasthan	Large blue flower, bold-seeded, high omega 3(58.20), high oil content (42%), light-brown-seeded, resistant to rust, powdery mildew, and bud fly
58	MAU AZAD ALSI-2 (LMS 149-4)	2011 2326 E, 10 October 2011	KL 178 x Hira	Manranipur (U.P.)	105–110	815 (R)	40.17	CG, Maharashtra, Karnataka, AP, Odisha	Medium white flower, medium- and brown-seeded, resistant to rust
59	NDL 2004-05	2011	Garima x RL 993	Faizabaad (U.P.)	125–130	1800(I)	40.0	Eastern Uttar Pradesh	Light flower, medium- and dark-brown-seeded, resistant to Alternaria blight, rust, and powdery mildew
60	NDL 2002	2011	Garima x EC 44	Faizabaad (U.P.)	130–135	1600 (I & R)	39.3	Eastern Uttar Pradesh	Light-blue flower, medium- and dark-brown-seeded, resistant to rust and powdery mildew and tolerant to bud fly
61	Pratap Alsi 2 (RL 26016)	2012 268 E, 28 January 2015	RL 914 x NL 93	Kota (Raj.)	129–135	1957 (I)	41.81	Rajasthan	Blue flower, shining-brown- and bold-seeded, powdery mildew, wilt, and bud fly. Moderately resistant to Alternaria blight, powdery mildew, wilt, and bud fly
62	PKVNL-260	2015 112 E, 12 January 2015	R 552 x RLC6	Nagpur (M.S.)	102–106	963 (R)	37.67	Maharashtra	Light flower, brown-seeded, moderately resistant to powdery mildew and bud fly
63	Kota Barani Alsi-3 (RL-292002)	2015	RL 903 x Ayogi	Kota (Raj.)	119–124	1370 (R)	38.73	Rajasthan	Blue flower, shining dark-brown-seeded, resistant to rust and moderately resistant to altarnaria blight, powdery mildew, and bud fly
64	Kota Barani Alsi-4 (RL-10193)	2015	Triveni x RL-1011	Kota (Raj.)	120–126	1100 (R)	40.37	UP, MP, Rajasthan	White flower, shining dark-brown-seeded, moderately resistant to altarnaria blight and powdery mildew
65	ChattisgadhAlsi-1 (RLC-133)	2015	NL-14 x ACC-26	Raipur (C.G.)	110–113	844 (R)	36.4	CG	White flower, early, moderately resistant to bud fly, altarnaria blight, powdery mildew, and rust
66	Tiara (JRF-2)	2015 1228 E	FT-889 x FT-895	CRIJEF (W.B.)	135–150	1290		H.P., Uttarakhand. Sikkim, A.P., U.P, Kashmir, and W.B.	White flower, tall, suitable for fibre yield
67	Arpita (OL 98-13-1)	2016 3540 E, 22 November 2016	RLC 29 x R 1871	Keonjhar (Odisha)	102–106	849 (R)	35.67	Odisha	Blue flower, light-brown-seeded, resistant to wilt and powdery mildew
68	UteraAlsi (RLC-143)	2016	LCK-88062 x T-397	Raipur (C.G.)	115–118	570 (U)	34.1	C.G., M.P., U.P., Jharkhand, Maharashtra, and Odisha	Suitable for Utera/para, blue flower, resistant to powdery mildew and rust, moderately resistant to budfly and altarnaria blight
69	Jawahar Linseed Sagar-79 (JLS-79)	2016 3540 E, 22 November 2016	Padmini x Laxmi-27	Sagar (M.P.)		1762(I)	40.35	Madhya Pradesh	Blue flower, suitable for irrigated conditions, moderately tolerant to powdery mildew, wilt, Alternaria blight, and bud fly
70	PKV-NL-260 (NL-260)	2016 3540 E, 2 November 2016	R552 x RLC-6	Nagpur (M.S.)	102–106	940(R)	35.56	Maharashtra	Blue flower, suitable for rainfed conditions, moderately tolerant to powdery mildew, wilt, Alternaria blight, and bud fly
71	DIVYA(BAU-06-03)	2016 3540 E, 22 November 2016	BAU-1008 x Kiran	Kanke (J.K.)	180	1538(I)	39.80	Punjab, Haryana, Himachal Pradesh, and Uttarakhand	White flower, resistant to rust and wilt
72	INDU (LCK 1108)	2016 1007 E, 30 March 2017	LMS1-2Kx LMS4-27	Kanpur (U.P.)	133–137	955 (I)	40.00	Whole of U.P.	Blue flower, bold- and brown-seeded, highly resistant to rust, resistant to alternaria blight and powdery mildew.
73	UMA (LCK-1101)	2016 1007 E, 30 March 2017	Polf 34 x Padmini	Kanpur (U.P.)	120–123	868 (R)	37.60	Whole of U.P.	Blue flower, medium- and light-brown-seeded, moderately resistant to rust, wilt, and Alternaria blight
74	VARSHA ALSI (RLC-148)	2017	SIKO 10 x Kiran	Raipur (C.G.)	110–114	1033 (R)	35.70	CG, MP, UP, JH, M.S., and Odisha	Blue flower, suitable for rainfed situation, moderately resistant to budfly, moderately susceptible to wilt and powdery mildew
75.	PRIYAM (BAU-2012-1)	2017 2805 E, 25 August 2017	BAU-2k-14 x Garima	Ranchi (Jharkhand)	173	1151(R)	37.15	Punjab, Haryana, H.P., and Uttarakhand.	White flower, dark brown seed
76.	JAWAHAR LINSEED SAGAR-95 (JLS-95)	2018 1379 E, 27 March 2018	JLS-27x GS-281	Sagar (M.P.)	133	1240(R)	39.40	Bundelkhand part of Uttar Pradesh, Raj. Madhya Pradesh, Karnataka, Odisha, Maharashtra, Chhattisgarh	White flower, bold-seeded, high omega 3 (53.50), high oil content (40%), suitable for rainfed and mechanical harvesting, resistant to rust, moderately resistant to wilt and powdery mildew, alternaria blight, and budfly
77	JAWAHAR LINSEED SAGAR-66 (JLS 66)	2018 399 E, 24 January 2018	Selection from Accn. 2511	Sagar (MP)	114	1200 (R)	40.50	Madhya Pradesh	Blue flower, suitable for rainfed conditions, bold-seeded, high omega 3 (55.96), high oil content (42%), resistant to rust, moderately resistant to wilt, powdery mildew, Alternaria blight, and budfly
78	SABOUR TISI-1 (BAUP-101)	2018 1379 E, 27 March 2018	Selection from Kishanganj local	Patna (Bihar)	124	453(U)	32.68	U.P., excluding Bundelkhand, Bihar, Jharkhand, W.B, and Assam	Blue flower, suitable for Utera, moderately resistant to rust and powdery mildew
80	HIM PALAM ALSI-2 (KL-263)	2018 1379 E, 27 March 2018	KL-223 x KL-224	Palampur	130	1633(I)	35.58	H.P., Haryana, and Jammu and Kashmir	Blue flower, suitable for rainfed conditions, moderately resistant to rust and powdery mildew
81	RAJAN	2018 1498 E, 1 April 2019	Gaurav x GS 234	Kanpur	130–135	1528(S) 1093(F)	38.01	Whole of U.P.	Blue flower, brown- and medium-seeded, highly resistant to rust, moderately resistant to alternaria blight
82	SURYA (LCK 1404)	2018 3220 E, 5 September 2019	Shubhra x H-25	Kanpur	155–160	1431(I)	35.49	H.P., Punjab, Haryana, and J&K	Blue flower, brown- and medium-seeded, highly resistant to rust, moderately resistant to wilt
83	UTERA ALSI-2 (RLC-153)	2019 1326 E, 2 April 2019	LCK88062x EC-1424	Raipur	118–120	519(U)	34.8	Assam, Bihar, Chhattisgarh, Jharkhand, Orrisa, M.P., M.H. and U.P.	Suitable for Utera conditions, moderately resistant to budfly
84	LSL-93	2019 3220 E, 5 September 2019	Selection from SLS-93	Latur	106	846(R)	37.07	Maharashtra	White flower, suitable for rainfed conditions, resistant to rust, moderately resistant to wilt, powdery mildew, alternaria blight, and bud fly
85	TL 99	2019 3220 E, 5 September 2019	RLC 6 X Solin	BARC Trombey	120	1274(I)	36.56	U.P., Bihar, Jharkhand, W.B., Assam, Nagaland	Low linolenic acid content (2–3%), edible oil purpose, resistant to wilt and rust, moderately resistant to budfly
86	JAWAHAR LINSEED 165	2019 3220 E, 5 September 2019	Meera x Polf 17	Hoshangabad (M.P.)	125	1392(I)	33.84	H.P, Punjab, and Jammu and Kashmir	Blue flower, suitable for irrigated conditions, resistant to rust and moderately resistant to powdery mildew, wilt, and bud fly
87	KOTA BARANI ALSI 5 (RL 29005)	2020 99 E, 9 November 2020	PKDL 41 x Meera	Kota (Raj.)	115–120	1150(R)	36.00	Rajasthan	Moderately resistant to wilt, alternaria blight, and bud fly
88	KOTABARANI ALSI 6 (RL 15584)	2020 3482 E, 7 October 2020	RL 101 x RLU 6	Kota (Raj.)	115–120	1200(R)	32.00	H.P, Punjab, and Jammu and Kashmir	Moderately resistant to wilt, powdery mildew, rust, alternaria blight, and bud fly
89	KOTA ALSI 6 (RL 13165)	2020 3482 E, 7 October 2020	RLC 25127 x RL 26016	Kota (Raj.)		1200(R)	32.00	UP, Jharkhand, Bihar, W.B., and Assam	Moderately resistant to wilt and powdery mildew, rust, alternaria blight, and bud fly
90	RLC 161 (SUVEE)	2020 3482 E, 7 October 2020	Ayogi x GS 234	Raipur (C.G.)		1262(R)	32.10	H.P, Punjab, and Jammu	Moderately resistant to alternaria blight and budfly
92	RLC 164	2020 500 E, 29 January 2021	Polf 22 x JRF 5	Raipur (C.G.)		1161(R)	32.60	H.P, Punjab and Jammu	Resistant to rust, moderately resistant to alternaria blight and bud fly
93	RLC 167	2020 500 E, 29 January 2021	T 397 x Polf 22	Raipur (C.G.)		1297(R)	34.20	H.P, Punjab and Jammu	Moderately resistant to wilt, powdery mildew, and budfly
93	APARNA (LCK1611)	2020 500 E, 29 January 2021	BAU610 x Parvati	Kanpur (U.P.)		1342(I)	33.00	H.P, Punjab, and Jammu	Resistant to Alternaria blight, moderately resistant to wilt and powdery mildew
95	BUAT ALSI-4 (LMS 15-31)	2020 500 E, 29 January 2021	P 18 x Accn. No. 2299	Mauranipur (U.P.)		1271(I)	31.80	Bundelkhand part of U.P., M.P., MH, K.N., Orisha, C.G.	Moderately resistant to powdery mildew and budfly
96	BIRSA TISI 1 (BAU 15-03)	2021 8 E, 24 December 2021	(Shekhar x RLC 80) x Garima	Ranchi, Jharkhand	128–130	1141(R)	41.80	Jharkhand	Resistant to wilt and powdery mildew, moderately resistant to alternaria blight and bud fly
97	PRIYAM (BAU 12-1)	2021 8 E, 24 December 2021	BAU 2K-1 x Garima	Ranchi, Jharkhand	128–130	1253(R)	40.70	H.P., Punjab, and Jammu and Jharkhand	Resistant to rust, wilt, alternaria blight, and powdery mildew, moderately resistant to bud fly
98	DIVYA (BAU 06-3)	2021 8 E, 24 December 2021	BAU 1008 x Kiran	Ranchi, Jharkhand	128–130	1538(I)	39.80	H.P., Punjab, Jammu, and Jharkhand	Resistant to powdery mildew and rust, moderately resistant to alternaria blight and wilt
99	SHUATS ALSI 2 (SHA-2)	2021 8 E, 24 December 2021	LCK 8879 x CI 1399	Naini (U.P.)	123–125	1110(R)	37.40	Uttar Pradesh	Resistant to powdery mildew and wilt, moderately resistant to rust
100	SABOUR TISI 2 (BRLS-101)	2021 8 E, 24 December 2021	SLS 72 x Shekhar	Sabour, Bihar	118	547(U)	38.20	U.P., Bihar, Jharkhand, W.B., Assam, M.P., M.H., C.G., Nagaland, Raj., Odisha, Karnataka	Resistant to rust and wilt, moderately resistant to Alternaria blight
101	SABOUR TISI 3 (BRLS-107-1)	2021 8 E, 24 December 2021	LCK 7035 x Shekhar	Sabour, Bihar	122	1883(I)	37.80	U.P., Bihar, Jharkhand, W.B., Assam, M.P., Nagaland, Raj., M.H., C.G., Odisha, Karnataka	Resistant to powdery mildew and wilt, moderately resistant to rust
102	(RLC 171)	2022 3254 E, 27 July 2022	Polf 22 x JRF 22	Raipur (C.G.)	132	1073(R)	34.53	Assam, Bihar, C.G., H.P., Jammu, JK, K.N., M.P., M.H., Nagaland, Orrisa, Punjab, Raj., and U.P.	Blue flower, moderately resistant to rust and bud fly
103	SABOUR TISI 4 (BRLS 121)	2023 1056 E, 6 March 2023	(NL 260 x Shekhar) x PKDL 71	Sabour, Bihar	120	1000	34.00	UP., Bihar, Jharkhand, W.B., Assam and Nagaland	Blue flower, moderately resistant to rust and bud fly
104	JAWAHAR LINSEED SAGAR 122 (JLS 122)	2023 1056 E, 6 March 2023	JLS 73 x JLS 66	Sagar M.P.	116	964(R)	41.00	Madhya Pradesh	Blue flower, semi-dehiscent capsule suitable for mechanical harvesting, bold-seeded (8 g), high omega 3 (54.20), resistant to rust, moderately resistant to powdery mildew, Alternaria blight, and bud fly
105	AZAD PRAGYA (LCK 1516)	1056 E, 6 March 2023	Shubhra x Shikha	Kanpur (U.P.)	128	1345(I)	35.00	Uttar Pradesh	Blue flower, resistant to rust and powdery mildew, moderately resistant to wilt and bud fly
106	SHUATS ALSI 5 (SHA-5)	2023 1056 E, 6 March 2023	RL 10205 x IC 564620	Naini (U.P.)	125–128	1250(I)	35.80	Uttar Pradesh	Blue flower, resistant to rust and powdery mildew, moderately resistant to wilt and bud fly

**Table 4:** Genome-wide association studies discovered quantitative trait nucleotides/loci for key abiotic stressors [196].

Trait	Candidate Gene	Type	QTN/QTL	Function	Reference
Root surface area stability	Lus10034840	Calcium transporting ATPase9, Plasma membrane type	Lu5-4,774,423	Pollen development	[82]
	Lus10016017	Catalase isozyme 3	Lu6-15,939,492	Response to water deprivation, promotion of drought stress tolerance	[81]
Total root length stability	Lus10039723	IAA amidosynthetaseGH3.6	Lus-20,209,630	Response to stress and root development	[82]
	Lus10039747	Diacylglycerol kinase 5		Cold and drought stress tolerance	[81]
	Lus10021019	Allene oxide synthase	Lu6-19,733,117	Stomatal closure and drought tolerance	[180]
	Lus10020997	S/Tproteinkinase SRK2E		Response to water deprivation and regulation of stomatal closure	[81]
Stress tolerance index	Lus10019811	Probable cinnamyl alcohol dehydrogenase1	Lu6-17,376,408	Drought tolerance	[82]
	Lus10014978	aquaporinPIP2-2	Lu14-23,517,150	Drought tolerance	[180]
	Lus10019781	L-Ascorbate peroxidase		Enhanced salt tolerance, drought tolerance, and cold tolerance	[181]
Total root volume stability	Lus10016017	Catalase Isozyme C	Lu6-15,961,789	Promotion of drought stress tolerance and response to water deprivation	[81]
Bundle weight under drought stress	Lus10040333	3-Ketoacyl-CoA synthase 19	Chr9:4203006	Drought tolerance and biomass-related traits	[217]
Canopy temperature under drought stress	Lus10013240	Xyloglucan endotransglucosylase/hydrolase protein 27	Chr2:23123754	Leaf size and veins and drought susceptibility index	[82]
	Lus10019365	Stromal cell-derived factor 2	Chr3:9279281	Heat stress and better stress tolerance indices	[217]
	Lus10024816	Cytochrome	Chr9:18937269	Moisture stress tolerance	[81]
Seeds per boll	Lus10021766	Mitogen-activated protein kinase kinasekinase 5	Chr9:15446958	Drought susceptibility index	[81]
Grain yield	Lus10042229	CBL-interacting protein kinase	Chr11:3972867	Drought	[180]
	Lus10042231	Translocon chloroplast 110		Heat shock and drought susceptibility index	[181]
Thousand-seed weight under drought stress	Lus10029127	Kelchrepeat F-box	Chr1:7029139	Ovule development and stress tolerance index	[82]
	Lus10029115	Ribosomal pentatricopeptide repeat protein 4		Seed development and stress tolerance	[82]
	Lus10030137	Nuclear factor Y subunit A1	Chr12:10910146	Seed development and drought stress tolerance	[81]
	Lus10030142	Translocated promoter region, nuclear pore anchor		Flowering, auxin signalling	[81]
Plant height under drought stress	Lus10029690/1	Cellulose synthase interactive	Chr5:1375386	Flax fibre and stress tolerance index	[82]
Yield	Lus10031398	Inositol Monophosphatase 1	Chr12:20557728	Drought tolerance	[81]

**Table 5:** Genome-wide association studies that discovered quantitative trait nucleotides/loci for key biotic stressors [196].

Trait	QTN/QTL	Candidate Gene	Type	Function	References
Pasmo resistance	QTL 45	Lus10031043	LRR receptor kinase	Bacterial pathogen-associated molecular pattern	[211]
				(PAMP) receptor	
		Lus10031058	Elongation Factor	Effector-trigger immunity	[135]
Fusarium wilt resistance	afB13	--		--	[218]
Powdery mildew resistance	QPM-crc-LG1	--		--	[219]
	-	Pm1			[135]
	Lu4-12,432,479	Lus10036891		RGA (WRKY transcription factor)	[212]

**Table 6:** Global transcriptome study of gene expression patterns in flax in response to significant abiotic and biotic stressors [196].

Trait	Tissue	DEGs/DEUs	Key Points	Platform/Tool	References
Response to salt stress	Flaxseeds	77,361,566 in neutral-salt stress, alkaline-salt stress, and alkaline stress	Photosynthesis-, pathogen-, and wax-related genes	Illumina HiSeq 2000	[238]
	Flaxseeds	3374 upregulated and 18,040 downregulated	Provide high-impact gene expression profile	Illumina high-throughput sequencing	[232]
Response to drought stress	Seeds, roots, and shoots	183:72 upregulated and 111 downregulated	Maintain homeostasis and growth	Combi matrix 90 K array	[72]
Response to drought-sensitive and tolerant varieties	Flax leaves	In cv.Z1413,245 upregulated and 4167 downregulated	DNA repair from ROS damage and proline biosynthesis	PacBIo ISO-seq	[239]
Response to osmotic stress under PEG-induced condition	Flaxseeds	3922:1487 upregulated and 2432 downregulated	Signal transduction and biochemical pathway	Illumina platform	[240]
Response to aluminium stress and high soil acidity	Flax seedlings	-	Compartmentalisation of Ca2+ in vacuoles	Illumina Platform	[230]
Response against flax rust *(Melanosporalini*)	Leaf tissues	-	Gene encoding PR protein and hydrolysis and uptake of nutrients	Illumina genome analyzer II	[241]
Response to Fusarium wilt	Roots, stem		Transduction and reception of pathogen signals	Illumina HiSeq 2000	[242]

**Table 7:** Important genes in flax that are activated and deactivated in response to biotic and abiotic stressors.

Character	Upregulated/Downregulated Genes	References
Salinity and alkalinity	HSP70 NAC family members, ABA, WRKY, PrxR	[238]
	LUS-miRNAs and miRNA-targeted genes	[281]
	UBE2 gene, mitochondrial termination factor family protein and auxin signalling F-box, Myb domain protein	[282]
Drought	Lipid transferase protein, cell wall synthesis genes, RUBISCO, PS1, r2r3-MYB transcription factor, EF-tu, LEA5, cytochrome P450 family proteins, brassinosteroid insensitive I-associated receptor kinase1, and AP2/ERF domain-containing transcription factor	[72]
	NAC domain protein	[112]
Heat	HSF	[283]
	Heat shock factors (HSFs)	[111]
	miRNAs and phasiRNAs	[114]
	GUS activity in sepals, petals, and pistils	[110]
Nutrient stress	WRKY, ING1 family JAZ and HARB11	[284]
Aluminium stress	miR319, UDP-glycosyl-transferasemiR390, glutathione-S-transferase and miR393	[234]
Rust	Avrs and CWDEs	[240]
Fusarium wilt	PAL, PCBER, SRG1, UGT73C3, AAA-ATPase ASD, mitochondrial (AATPA), glucan endo-1,3-β-glucosidase, MYB transcription factors, ERD dehydrins, and auxin-responsive protein SAUR, WKY3, WRKY70, WRKY75, MYB113, and MYB108	[234,285]
Fusarium culmorum	PAL, CCR, CAD, UGT, and TD	[233]

## Data Availability

Not applicable.
